# Cell-Based Small-Molecule Screening Identifying Proteostasis Regulators Enhancing Factor VIII Missense Mutant Secretion

**DOI:** 10.3390/biom15040458

**Published:** 2025-03-21

**Authors:** Vishal Srivastava, Zhigang Liu, Wei Wei, Yuan Zhang, James C. Paton, Adrienne W. Paton, Tingwei Mu, Bin Zhang

**Affiliations:** 1 Genomic Medicine Institute, Cleveland Clinic Lerner Research Institute, Cleveland, OH 44195, USA; srivasv@ccf.org (V.S.); liuz9@ccf.org (Z.L.); yuanzhang@whu.edu.cn (Y.Z.); 2 Research Centre for Infectious Diseases, Department of Molecular and Biomedical Science, University of Adelaide, Adelaide, SA 5005, Australia; james.paton@adelaide.edu.au (J.C.P.); adrienne.paton@adelaide.edu.au (A.W.P.); 3 Department of Physiology and Biophysics, Case Western Reserve University School of Medicine, Cleveland, OH 44106, USA; txm210@case.edu; 4 Department of Molecular Medicine, Cleveland Clinic Lerner College of Medicine of Case Western Reserve University School of Medicine, Cleveland, OH 44195, USA

**Keywords:** factor VIII, secretion, Vorinostat, proteostasis regulators, BiP/GRP78

## Abstract

Missense mutations are the most prevalent alterations in genetic disorders such as hemophilia A (HA), which results from coagulation factor VIII (FVIII) deficiencies. These mutations disrupt protein biosynthesis, folding, secretion, and function. Current treatments for HA are extremely expensive and inconvenient for patients. Small molecule drugs offer a promising alternative or adjunctive strategy due to their lower cost and ease of administration, enhancing accessibility and patient compliance. By screening drug/chemical libraries with cells stably expressing FVIII–Gaussia luciferase fusion proteins, we identified compounds that enhance FVIII secretion and activity. Among these, suberoylanilide hydroxamic acid (SAHA) improved the secretion and activity of wild-type FVIII and common HA-associated missense mutants, especially mild and moderate ones. SAHA increased FVIII interaction with the endoplasmic reticulum chaperone BiP/GRP78 but not with calreticulin. Lowering cellular BiP levels decreased SAHA-induced FVIII secretion and enhancing BiP expression increased FVIII secretion. SAHA also enhanced secretion and BiP interactions with individual domains of FVIII. In vivo, treating mice with SAHA or a BiP activator boosted endogenous FVIII activity. These findings suggest that SAHA serves as a proteostasis regulator, providing a novel therapeutic approach to improve the secretion and functionality of FVIII missense mutants prone to misfolding.

## 1. Introduction

Hemophilia A (HA) is a bleeding disorder caused by a deficiency of coagulation factor VIII (FVIII), resulting in life-threatening bleeding in severe cases. HA affects approximately 1 in 5000 male births worldwide [1,2]. FVIII is an essential cofactor for the serine protease factor IX (FIX), which activates factor X (FX) in the blood coagulation cascade [3]. Based on FVIII activity levels, HA is categorized as severe (<1% of normal activity), moderate (1–5%), or mild (5–40%), with symptom severity correlating to residual FVIII activity [1]. Current treatments include FVIII protein replacement and bi-specific antibody (emicizumab) therapies [4]. However, these therapies are prohibitively expensive, limiting access even in wealthy nations and making them largely inaccessible in lower-income countries [5]. Additionally, they often require frequent office visits for infusions, which can pose compliance challenges for patients. Small-molecule drugs offer an attractive alternative or supplemental treatment due to their lower costs, which improve accessibility, and ease of administration, which enhances patient compliance [6]. Currently, the only available small-molecule treatment is desmopressin (DDAVP), which temporarily increases plasma FVIII levels in patients with mild to moderate HA by stimulating the release of FVIII stored in the Weibel–Palade bodies of endothelial cells [7,8].

FVIII is a large glycoprotein that relies on endoplasmic reticulum (ER) chaperones for proper folding and secretion [9]. Key chaperones involved in this process include protein disulfide isomerases (PDIs) for disulfide bond formation, immunoglobulin binding protein (BiP/GRP78), calnexin (CANX), and calreticulin (CALR) for protein folding and quality control [10,11]. Correctly folded FVIII interacts with the LMAN1-MCFD2 cargo receptor complex and is transported to the Golgi apparatus in coat protein complex II (COPII)-coated vesicles [12,13]. However, even wild-type (WT) FVIII is inherently prone to misfolding [14,15]. Misfolded FVIII can activate the unfolded protein response (UPR), an adaptive pathway designed to alleviate ER stress by resolving protein misfolding [16,17]. If misfolding persists, terminally misfolded proteins are targeted for elimination via the ER-associated degradation (ERAD) pathway [11]. Prolonged UPR activation due to the accumulation of misfolded FVIII may result in apoptosis [9].

Proteostasis encompasses the cellular processes that maintain protein homeostasis, including protein biogenesis, folding, trafficking, and degradation [18,19]. Disturbances in proteostasis are a prominent feature in the pathogenesis of missense mutations, which are most common in HA [1,2]. Missense mutations usually do not affect expression levels, but they can impair protein biosynthesis, folding, secretion, and function [20,21,22,23,24,25]. Proteostasis regulators (PRs) are small molecules designed to enhance the proteostasis network’s capacity by targeting various steps in protein synthesis, degradation, and secretion pathways. PRs are being developed for several diseases, including cystic fibrosis, lysosomal storage diseases, and neurological disorders [26,27,28]. Despite their potential, limited research has focused on using PRs to rescue FVIII defects. Many FVIII missense mutations retain partial function, particularly those associated with mild to moderate HA [20,21,22,23,24,25]. Enhancing the folding and secretion of these mutants could alleviate ER stress, increase plasma FVIII activity, and mitigate symptoms. In this study, we screened compound libraries to identify potential PRs that enhance the secretion and function of misfolding-prone FVIII mutants and investigated the underlying molecular mechanisms.

## 2. Materials and Methods

### 2.1. Cell Line, Bacterial Strains, Reagents, and Antibodies

HEK 293 (CRL-1573 from American Type Culture Collection, Manassas, VA, USA) and HEK 293T (CRL-3216, ATCC) cells were used for the stable and transient expression of FVIII and were cultured in Dulbecco’s Modified Eagle’s Medium (DMEM), supplemented with 10% Fetal Bovine Serum (FBS) and 2mM L-glutamine. DMEM, FBS, and media supplements were purchased from ThermoFisher (Waltham, MA, USA). XL10-Gold ultra-competent and DH5α competent cells were used for site-directed mutagenesis and routine cloning, respectively. Restriction enzymes, T4 DNA ligase, and Phusion high-fidelity DNA polymerase were purchased from New England Biolabs (Ipswich, MA, USA). All chemical reagents used in this study were of analytic grade and were procured from Millipore Sigma (St. Louis, MO, USA) and Cayman Chemical (Ann Arbor, MI, USA). Rat anti-GRP78 and mouse anti-BiP/GRP78 antibodies were purchased from BD Biosciences (Franklin Lakes, NJ, USA, 610979) and Santa Cruz (Dallas, TX, USA, sc-13539). Mouse anti-human calreticulin was purchased from Santa Cruz (sc-166837) and rabbit anti-calnexin from Millipore Sigma (SAB4503258). Mouse anti-β-actin and rabbit and mouse anti-FLAG (A5441, F7425, F1804) were purchased from Millipore Sigma.

### 2.2. Establishment of Cell Lines Stably Expressing B-Domain-Deleted FVIII–Gaussia Luciferase Fusion

**Figure 1:**
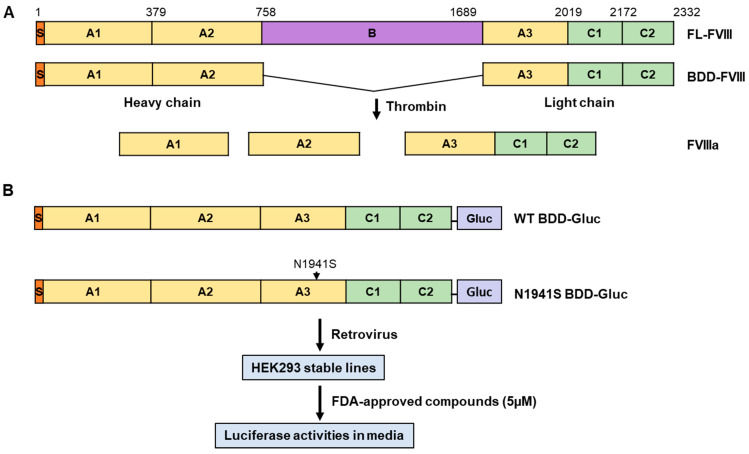
FVIII domain structures and screening of compounds that enhance FVIII mutant secretion. (**A**) The heavy chain comprises the A1 and A2 domains, while the light chain includes the A3, C1, and C2 domains. The B domain in FL-FVIII, dispensable for FVIII function, is cleaved during activation. Both FL-FVIII and BDD-FVIII form the same three-subunit activated FVIII (FVIIIa) after thrombin activation. (**B**) HEK293 cells stably expressing WT BDD-Gluc or the p.N1941S mutant BDD-Gluc were treated with compounds from the FDA-approved compound library. FVIII fusion protein secretion was quantified by measuring luciferase activity in the culture media.

Human cDNA encoding B domain-deleted FVIII (BDD-FVIII) was fused in-frame with humanized Gaussia luciferase (Gluc) cDNA [29], separated by a short linker sequence (AAAGGGGS) as described previously [30] (Figure 1). The resulting fusion gene (BDD-Gluc) was cloned into the retroviral vector pMSCV. Missense mutations were introduced into the WT BDD-Gluc construct using the QuikChange II Site-Directed Mutagenesis Kit (Agilent Technologies, Santa Clara, CA). All constructs containing mutations were validated by Sanger DNA sequencing to ensure the presence of the intended mutation and the absence of unintended changes. Retroviral particles were generated in HEK 293T cells and used to transduce HEK 293 cells. The infected cells were selected with 2 μg/mL puromycin for at least 10 days and maintained in 1 μg/mL puromycin as stable expression cell lines.

### 2.3. Construction of Plasmids Expressing Full-Length FVIII and Individual FVIII Domains with Missense Mutations

To construct full-length FVIII (FL-FVIII) with missense mutations, selected missense mutations were introduced into the pMT2-FVIII expression vector [25] using site-directed mutagenesis. To verify the presence of the intended mutation and the absence of unintended changes, Sanger sequencing was performed for the relevant region of the F8 cDNA. To further ensure no off-target modifications in the remaining F8 cDNA or vector DNA, the mutation-containing segment was excised as one of three fragments (XhoI-KpnI, KpnI-EcoRV, or EcoRV-SalI) and used to replace the corresponding fragment in the WT pMT2-FVIII vector. Missense mutations were also introduced into individual domains of FVIII (with N-terminal signal sequences and FLAG tags) cloned into either pED (A2) or pZ (C) expression vectors [24,25]. FVIII expression plasmids were transfected into HEK 293T cells using FuGENE6 (Promega, Madison, WI, USA) [30].

### 2.4. High-Throughput and Secondary Screening Using a Secreted Luciferase Assay

A high-throughput screening (HTS) approach was employed to identify compounds that enhance FVIII activity. HEK293 cells stably expressing BDD-Gluc were treated with small molecules from an FDA-approved compound library with 978 compounds (Selleckchem, Houston, TX, USA) and an in-house natural compound library with 480 plant-derived compounds. Compounds were prepared in dimethyl sulfoxide (DMSO) at a stock concentration of 10 mM and stored at −80 °C in 96-well plates. HEK293 cells (5 × 10^4^ cells/mL) stably expressing BDD-Gluc were seeded in 96-well plates in triplicate. After 16 h, the cells were treated with fresh media containing test compounds (5 µM) or DMSO (control) for 24 or 48 h. Subsequently, 20 µL of culture media was transferred to black 96-well plates and assayed for Gluc activity. The assay utilized 50 µL of 10 µM coelenterazine (GoldBio, St. Louis, MO, USA) in 0.1 M Tris (pH 7.4) and 0.3 M sodium ascorbate, measured using an LMAX II384 luminometer (Molecular Devices, San Jose, CA, USA), as described previously [30]. HTS data were analyzed using the screening window coefficient (Z-score) to evaluate each compound’s effect on relative luminescence. The Z-score was calculated as Z = (x − μ)/σ, where *x* represents the average relative luminescence after drug treatment, *μ* denotes the control’s average luminescence, and σ is the standard deviation of the control. Candidate compounds identified in the primary screening were further tested for the secondary screening. HEK293 cells stably expressing BDD-Gluc were treated with the selected compounds for 24 or 48 h. Gluc activity in the culture media was measured as described above.

### 2.5. FVIII Antigen and Activity Assays

FVIII protein levels in cell culture media and mouse plasma were measured by a matched-pair antibody set (Affinity Biologicals, Ancaster, ON, Canada) for ELISA of human FVIII antigen using the standard protocol. Cell culture media were tested for FVIII activity using the Chromogenix Coatest^®^ SP4 FVIII assay kit (DiaPharma, West Chester, OH, USA). Pooled normal human plasma (George King Bio-Medical, Overland Park, KS, USA) was used to generate standard curves.

### 2.6. Immunoblotting and Immunoprecipitation

Cells were lysed in the Nonet P-40 lysis buffer (NP-40 lysis buffer: 50 mM Tris-Cl pH 7.5, 150 mM NaCl, 1% Nonet P-40, 0.05% SDS) with a protease inhibitor cocktail (Roche, Basel, Switzerland). A monoclonal mouse anti-human FVIII antibody [31] was conjugated to agarose beads using the AminoLink plus immobilization kit (ThermoFisher) and then used in immunoprecipitation, as described in [25]. The immunoblotting protocol was described recently [24].

### 2.7. Animal Study

The animal experimental protocols were approved by the Institutional Animal Care and Use Committee (IACUC) of Cleveland Clinic Lerner Research Institute. Suberoylanilide hydroxamic acid (SAHA, 100 mg/kg) and BiP Inducer X (BIX, 20 mg/kg) were given to 4-week-old male CD-1 mice by intraperitoneal (IP) injection for 3 constitutive days. On day 4, the mice were anesthetized with 50 µg/kg of sodium pentobarbital (Sagent, Schaumburg, IL, USA), and blood was collected in 4% sodium citrate anticoagulant by cardiac puncture. Blood was centrifuged twice at 7500 rpm for 10 min in a microfuge. Plasma was aliquoted and stored at −80 °C until use.

### 2.8. Statistical Analysis

Statistics were calculated and plotted using Microsoft Excel or GraphPad Prism Version 10. ANOVA with a Tukey post hoc test or a two-tailed Student’s *t*-test was used for the comparison of continuous variables, as appropriate. All *p* values were two-sided, and *p* < 0.05 was considered statistically significant.

## 3. Results

### 3.1. Screening of FDA-Approved Compound Library to Identify Compounds That Enhance FVIII Secretion

**Figure 2:**
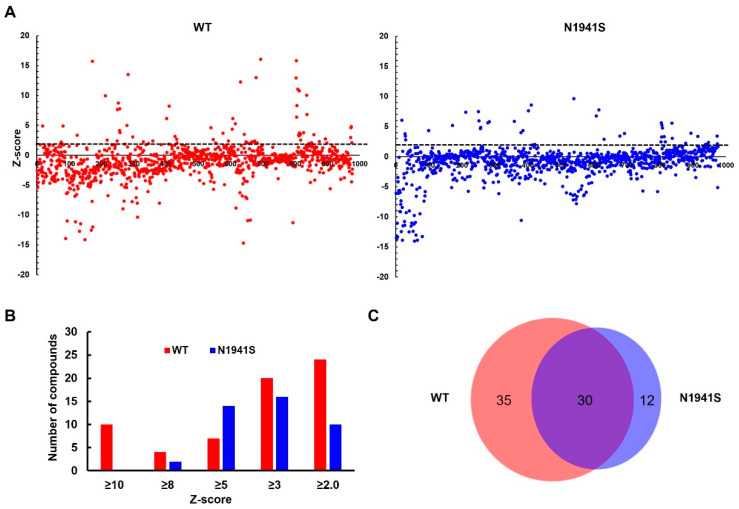
High-throughput screening of 978 FDA-approved compounds. (**A**) Z-scores for each compound were calculated based on the effects on WT and p.N1941S BDD-Gluc secretion. The *X*-axis represents the cumulative number of compounds screened, with each dot corresponding to one compound. Compounds with Z-scores ≥ 2.0, indicated by the black dashed line, were subjected to secondary screening. (**B**) Bar graph showing the distribution of compounds based on Z-scores for WT (red bars) and p.N1941S (blue bars) BDD-Gluc. (**C**) Venn diagram illustrating the overlap of compounds with Z-scores ≥ 2.0 between the WT and p.N1941S cell lines.

FL-FVIII has the domain structure of A1-A2-B-A3-C1-C2, where the B domain is dispensable for the cofactor function (Figure 1A). We developed a high-throughput Gluc-based assay to screen for compounds that enhance the secretion of WT and an FVIII missense mutant. In this assay, either BDD-FVIII or BDD-FVIII with a missense mutation (p.N1941S) was fused at its C-terminus with a humanized Gluc and stably expressed from a retrovirus construct in HEK293 cells (Figure 1B). The p.N1941S mutation was chosen because the mutant was previously reported to be functional, and the major defect was domain-specific misfolding resulting in decreased secretion [22]. Gluc fusion provides a tag for detecting FVIII fusion protein secreted from cells and a rapid assay for drug screening. In the primary screening, 978 FDA-approved compounds were screened against the WT and the p.N1941S mutant cell lines (Figure 1B). The cells were treated with 5 µM of compounds for 24 h, and the cell media were tested for luciferase activity (Figure 1). Increased and decreased relative luciferase unit (RLU) in the cell media showed a positive effect on FVIII secretion, while decreased RLU showed either a negative effect on FVIII secretion or cell viability. The RLU for each compound was converted into a Z-score. Primary screening data (Table S1) were plotted using a bubble scatter plot for 978 compounds for both WT and the p.N1941S mutant cell lines (Figure 2A). The primary screening identified 65 compounds with a Z-score ≥ 2 in the WT cell line (Figure 2B) and 42 compounds in the p.N1941S mutant cell line, with 30 compounds overlapping between the two tested cell lines (Figure 2C, Table S1).

**Figure 3:**
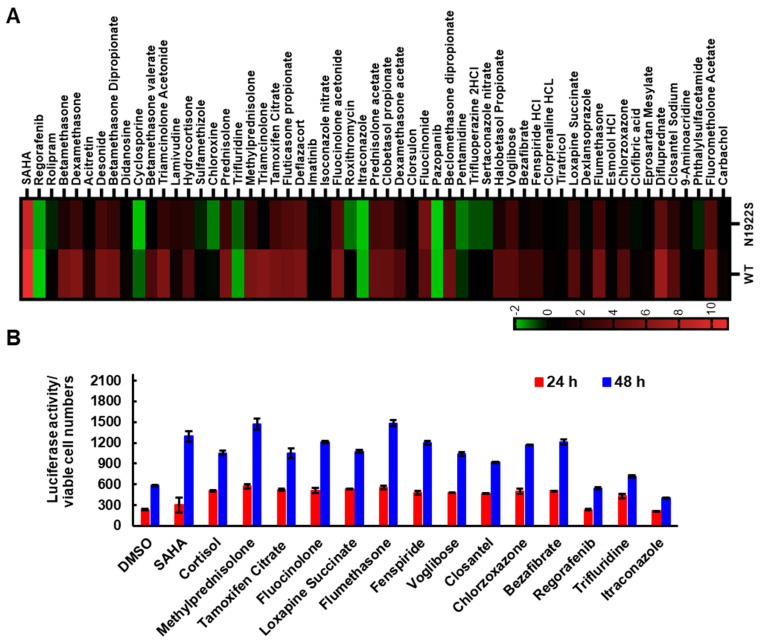
Secondary screening and validation of candidate compounds. (**A**) Fifty-seven compounds identified in the primary screening were subjected to secondary screening in the WT-BDD and p.N1941S cell lines. The heat map depicts the Z-scores of all 57 compounds. (**B**) Fifteen selective compounds were further validated for their ability to enhance or reduce WT BDD-Gluc secretion. Following treatment with each compound for 24 and 48 h, luciferase activity was measured in the media, normalized to 100,000 cells, and compared against DMSO-treated controls.

Based on the primary screening results, we selected 37 compounds with Z-scores ≥ 2.0, including the 30 compounds common between the two cell lines, for secondary screening. We also tested 20 compounds that cause decreased RLUs in one or both cell lines. Z-score values of all 57 compounds were presented as a heat map (Figure 3A). This secondary screening confirmed 30 compounds that increased RLUs and five compounds that decreased RLUs. Interestingly, 22 chemicals among the “increase” group were cortisol and its analogs. We also performed screening using an in-house natural compound library with 480 plant-derived compounds (Table S2). The two compounds that increased FVIII-Gluc secretion from this screening were cortisol and betamethasone dipropionate (Figure S1).

Next, we further validated 12 compounds in the “increase” group, including cortisol and three of its analogs, and three compounds in the “decrease” group by measuring Gluc fusion secretion after 24 and 48 h treatments, normalized to cell numbers. After the 48 h treatments, all compounds in the “increase” group exhibited luciferase activities exceeding 150% compared to the DMSO control (Figure 3B, Table S1). Only one of the three compounds in the “decrease” group showed a decrease in RLU over 30% (Figure 3B).

### 3.2. SAHA Significantly Enhanced the Secretion of FVIII Missense Mutants

**Figure 4:**
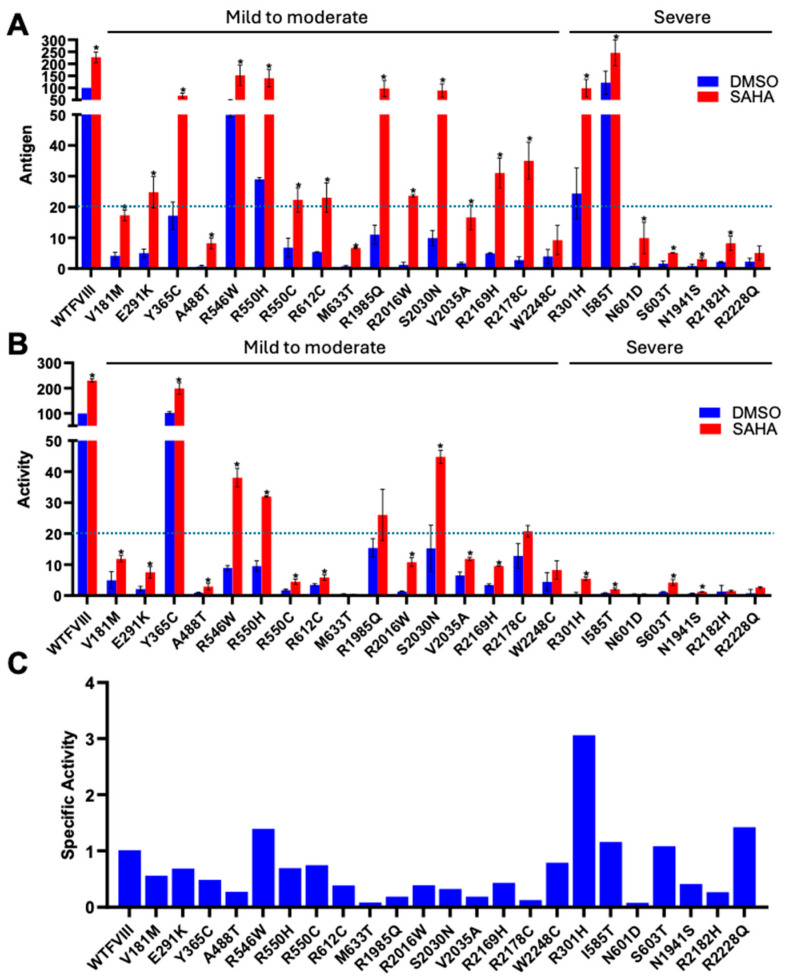
SAHA increases secreted FVIII activity and antigen levels of missense mutants. (**A**,**B**) HEK293T cells transiently transfected with WT FL-FVIII or 23 missense mutants were treated with 2.5 µM SAHA 24 h post-transfection. The culture media collected 24 h later were analyzed for FVIII activity and antigen levels, expressed as percentages relative to DMSO-treated WT FVIII. The p.Y365C mutant has functional defects that are not detected by our activity assay [36]. * *p* < 0.05. Data are presented as mean ± SD from three independent experiments. Dotted lines denote 20% of WT levels. (**C**) Specific activities of FL-FVIII missense mutants are calculated as the ratio of fold changes in FVIII activity to antigen levels. The dotted line represents the specific activity of WT FVIII.

One of the compounds identified was SAHA, also known as Vorinostat (Figure S2). SAHA has previously been shown to act as a PR for several other proteins, including α1-antitrypsin, CFTR, and neuroreceptors [32,33,34,35]. To further test the efficacy of SAHA, we introduced 23 missense mutations into FL-FVIII, including some of the most frequently reported ones in HA according to the Coagulation Factor Variant Databases (https://dbs.eahad.org/FVIII, accessed on 12 October 2024) (Table 1). These mutations are localized in different domains of FVIII. WT and mutant FVIII were expressed in HEK293T cells and treated with SAHA (2.5 µM) for 24 h. Cell media were collected to measure FVIII antigen and activity levels. SAHA significantly increased antigen levels in the cell media of WT FVIII and most FVIII missense mutants (Figure 4A). Thirteen mutants had antigen levels reaching >20% of WT FVIII after SAHA treatment (Figure 4A). Most mutants (16 out of 23) also exhibited significant increases in cofactor activity levels (Figure 4B). Although less effective, SAHA can also rescue some severe mutants. Four of the seven tested severe mutants showed significant increases in cofactor activities from <1% to 1–5%, which is the activity range of moderate HA (Figure 4B). Based on FVIII-specific activity (ratio of activity increase over antigen increase) data (Figure 4C), SAHA mostly increased FVIII secretion without proportionally increasing the activities, suggesting that most missense mutants have functional or folding defects that were not completely corrected by SAHA.

### 3.3. SAHA Increased the Interaction of FVIII with an ER Chaperone BiP

**Figure 5:**
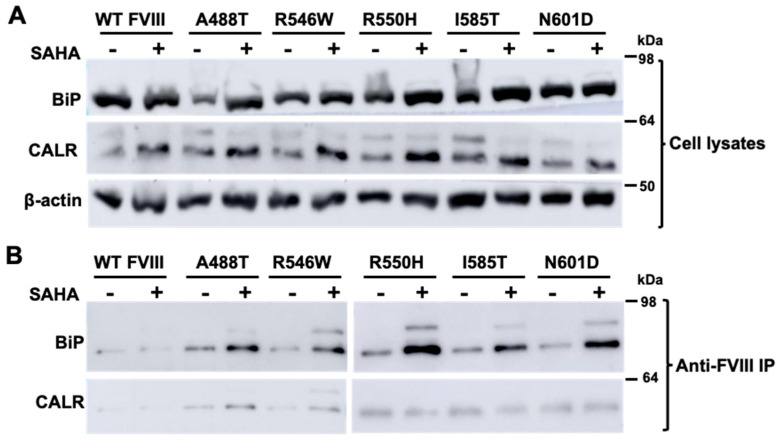
SAHA modulates BiP and CALR expression and their interactions with FVIII. HEK293T cells transiently transfected with WT FL-FVIII or the indicated missense mutants were treated with 2.5 µM SAHA 24 h post-transfection. (**A**) After 24 h treatments, cell lysates were analyzed by immunoblotting with antibodies against BiP, CALR, and β-actin. (**B**) FVIII complexes were immunoprecipitated with bead-conjugated anti-FVIII and immunoblotted with antibodies against BiP and CALR.

To explore the mechanism of SAHA on FVIII secretion, we examined interactions of FVIII with ER chaperones BiP and CALR. HEK293T cells were transfected with WT FL-FVIII and selected missense mutants (p.A488T, p.R546W, p.R550H, p.I585T, and p.N601D). SAHA treatment enhanced the expression of BiP and CALR in cells expressing WT or missense mutant FVIII (Figure 5A). We next examined the interactions of FVIII with BiP and CALR. This was achieved by co-immunoprecipitating BiP and CALR using bead-conjugated FVIII antibodies. Anti-FVIII beads pulled down both BiP and CALR (Figure 5B). Following SAHA treatment, the amounts of BiP that co-immunoprecipitated with missense mutant FVIII increased significantly (Figure 5B).

### 3.4. SAHA Increased the Secretion of Individual FVIII Subunits

**Figure 6:**
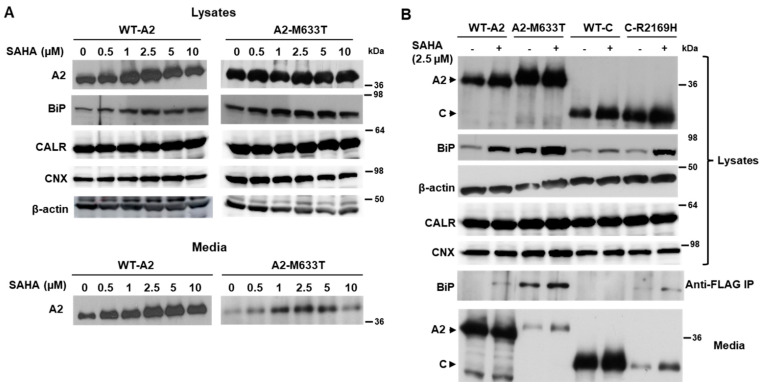
Effects of SAHA on the secretion and ER chaperone interactions of the FVIII A2 and C domains. (**A**) HEK293T cells transiently transfected with the FLAG-tagged A2 domain or A2 domain containing the M633T mutation were treated with varying concentrations of SAHA 24 h post-transfection. After 24 h of treatment, cell lysates were analyzed by immunoblotting with antibodies against the FLAG tag, BiP, CALR, CNX, and β-actin. Secreted A2 domain levels in culture media were detected by immunoblotting with an anti-FLAG antibody. (**B**) HEK293T cells transiently transfected with the FLAG-tagged A2 domain, C domain, A2 domain with the M633T mutation, or C domain with the R2169H mutation were treated 2.5 µM SAHA 24 h post-transfection. After 24 h of treatment, cell lysates were analyzed by immunoblotting with antibodies against the FLAG tag, BiP, CALR, CNX, and β-actin or subjected to immunoprecipitation with anti-FLAG followed by immunoblotting for BiP and CALR. Secreted levels of the A2 and C domains in culture media were detected by anti-FLAG immunoblotting.

Published reports indicate that the half maximal effective concentration (EC50) of SAHA ranges from 0.6 to 7.5 µM, depending on experimental conditions. Secretion of FL-FVIII reached the highest level at 2.5 µM (Figure S3A) and the estimated EC50 was ~1.86 µM (Figure S3B). We previously showed that the FLAG-tagged A2 and C domains of FVIII are readily secreted and missense mutants located in these domains decrease their secretion. We tested the responses of the WT A2 domain and the p.M633T mutant A2 domain to SAHA doses between 0 and 10 µM. The secretion of WT and the mutant A2 domain peaked at a SAHA dose of 2.5 µM (Figure 6A). Intracellular BiP levels increased upon SAHA treatments, also peaking at 2.5 µM (Figure 6A). Next, we treated cells expressing the A2 or C domain, as well as a missense mutant of each domain (p.M633T and p.R2169H), with 2.5 µM SAHA. Again, increased secretion was observed along with increased intracellular BiP levels (Figure 6B). Immunoprecipitation with the anti-FLAG antibody revealed an increased interaction of BiP with both mutant subunits (Figure 6B). Increased secretion was also demonstrated in a time-course study of both WT A2 and the A2-M633T mutant treated with 2.5 µM SAHA (Figure S4).

### 3.5. Decreasing BiP Levels Reversed SAHA-Mediated Stimulation of FVIII Secretion

**Figure 7:**
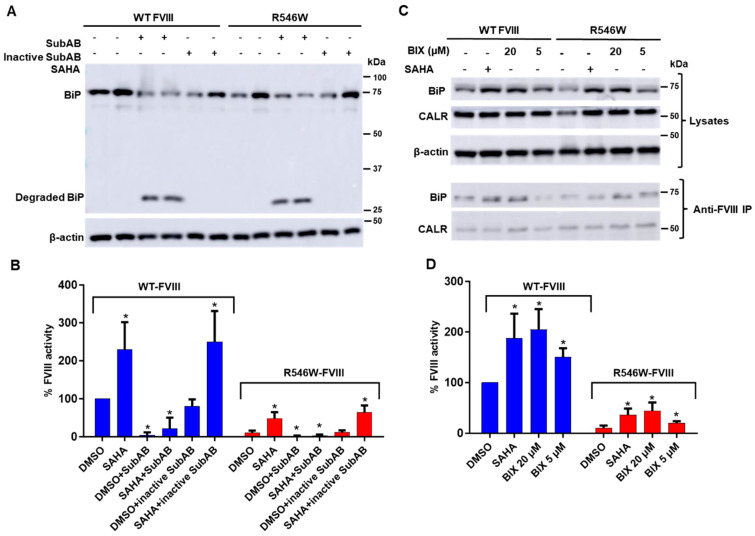
Effects of SubAB and BIX on BiP expression and FVIII secretion. (**A**) HEK293T cells were transfected with WT FL-FVIII or the R546W missense variant and treated with SAHA (2.5 µM), SubAB (0.2 µg/mL), or inactive SubAB (0.2 µg/mL). BiP expression was analyzed 24 h post-treatment via immunoblotting. (**B**) FVIII activity levels in the culture media were measured and expressed as percentages of untreated WT levels. Data are presented as the mean ± SD from three independent experiments. * *p* < 0.05. (**C**) HEK293T cells transfected with WT FL-FVIII or the R546W missense variant were treated with SAHA (2.5 µM) and BIX (20 µM and 5 µM) for 24 h. BiP and CALR expression levels were analyzed via immunoblotting. FVIII interactions with BiP and CALR were assessed by immunoprecipitation with bead-conjugated anti-FVIII, followed by immunoblotting for BiP and CALR. (**D**) FVIII activity levels in the culture media were measured and expressed as percentages of untreated WT levels. Data are presented as the mean ± SD from three independent experiments. * *p* < 0.05.

To understand the significance of the SAHA-induced increase in the interaction of BiP with FVIII, we used the subtilase cytotoxin SubAB to decrease BiP levels. As a BiP suppressor, SubAB cleaves and inactivates BiP in the ER, and BiP is the only identified target of SubAB [37]. Cells expressing WT or missense mutant FVIII were treated with SAHA along with SubAB and with an inactive variant of SubAB, and the impact on FVIII secretion was assessed by measuring the activities of FVIII secreted into the medium. Treatment with SubAB (0.2 µg/mL) reduced cellular BiP levels by proteolysis (note the degraded BiP fragment in SubAB-treated cells in Figure 7A) and decreased the secretion of both WT and the p.R546W mutant FVIII (Figure 7B). In the presence of SubAB, SAHA failed to increase FVIII secretion (Figure 7B). On the other hand, treatment with inactive SubAB (0.2 µg/mL) did not affect BiP expression (Figure 7A) and the secretion of WT and the p.R546W mutant FVIII (Figure 7B). Inactive SubAB also did not affect the SAHA-induced increase in the secretion of both WT and the p.R546W mutant FVIII (Figure 7B).

### 3.6. A BiP Activator Enhanced FVIII Secretion

BIX (1-(3, 4-dihydroxy-phenyl)-2-thiocyanate-ethanone) is a known activator of BiP. It induces BiP expression through an ATF6-dependent mechanism without substantially inducing the expression of other ATF6 target genes [38]. BIX at 20 µM increased BiP expression and the amounts of BiP that co-immunoprecipitated with FVIII (Figure 7C), similar to the effects of SAHA (Figure 7A). BIX (20 µM) also enhanced FVIII secretion, with FVIII activity in the cell medium increased to levels similar to SAHA (2.5 µM)-treated cells (Figure 7D). Enhanced secretion was also observed in the cells treated with a lower concentration (5 µM) of BIX (Figure 7D). Interestingly, no further increases in FVIII secretion were observed when SAHA and BIX were combined to treat the cells (Figure S5), indicating that BIX and SAHA share a common molecular mechanism to enhance FVIII expression and secretion by BiP upregulation.

### 3.7. SAHA and BIX Treatments Increase Endogenous FVIII Levels in Mice

**Figure 8:**
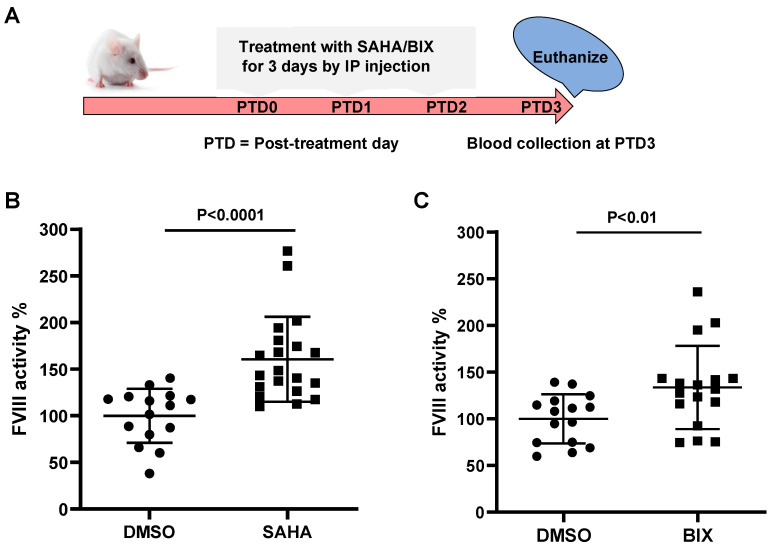
In vivo efficacy of SAHA and BIX on endogenous mouse FVIII. (**A**) CD-1 mice were treated with SAHA (100 mg/kg) or BIX (20 mg/kg) for three consecutive days. Plasma was collected via cardiac puncture on day 4 to measure endogenous FVIII activity. DMSO-treated mice served as the control group. (**B**) Endogenous FVIII activity levels in SAHA-treated mice compared to DMSO-treated controls. (**C**) Endogenous FVIII activity levels in BIX-treated mice compared to DMSO-treated controls. Data are presented as scatter plots, with each symbol representing an individual mouse. Horizontal bars indicate mean FVIII activity levels ± SD.

To explore the impact of SAHA and BIX on endogenous FVIII secretion in vivo, we treated the outbred male CD-1 mice with SAHA or BIX to determine their effects on plasma FVIII levels. SAHA, BIX, or DMSO controls were administered for 3 constitutive days by IP injection, and blood was collected on day 4 to measure plasma FVIII activity levels (Figure 8A). SAHA at 100 mg/kg increased endogenous FVIII activity levels by 1.60-fold compared to the DMSO controls (*n* = 15 in the DMSO group and *n* = 20 in the SAHA group, *p* < 0.0001) (Figure 8B). BIX at 20 mg/kg also enhanced plasma FVIII activity (by 1.33-fold) compared to the DMSO controls (*n* = 15 in the DMSO group and *n* = 17 in the BIX group, *p* < 0.01) (Figure 8C).

## 4. Discussion

In this study, we screened small-molecule libraries to identify PRs that enhance the secretion of WT FVIII and a missense mutant FVIII. The FDA-approved drug library is ideally suited for drug repurposing studies. Our screening is based on the sensitive Gluc assay that monitors changes in the amount of FVIII-Gluc fusion protein in cell culture medium upon treatments. Compounds that increase the amount of fusion protein secreted into the cell medium may act on different steps of the production and secretion of the FVIII-Gluc fusion protein. Interestingly, several compounds with the strongest effects are corticosteroids and their derivatives. Increased FVIII activity has been observed in patients treated with high doses of methylprednisolone [39,40,41]. Corticosteroids may increase the transcription of the *F8* gene. Among non-corticosteroids, we focused our studies on the mechanism of SAHA, which is a histone deacetylase inhibitor (HDACi) approved by the FDA to treat cutaneous T-cell lymphoma [42,43]. SAHA was previously shown to be a PR for other proteins by increasing the function of misfolded proteins [35,44,45]. Also, another HDACi (LB205) was shown to restore the function of the most prevalent mutations for non-neuronopathic (N370S) and neuronopathic (L444P) in Gaucher disease in cell culture and mouse models [46,47].

FVIII is a large glycoprotein that is known to be prone to misfolding in the ER [14,15] and to induce the UPR, so, unsurprisingly, the identified compounds increased the activities of both the WT and p.N1941S mutants. The observation that SAHA increased WT FVIII secretion suggests that the mechanism is not specific to individual mutation. The same molecular mechanism underlies the increased WT and missense mutant FVIII secretion. Indeed, when SAHA was used to treat a large number of missense mutants of FVIII, most responded with higher-secreted FVIII activity. In particular, SAHA is effective for those mutants that retain some secretion levels of the partially active protein, such as mild and moderate mutants. The observation that mild and moderate mutants respond well to PR treatment is important because mild and moderate diseases do not qualify for gene therapy trials and prophylactic factor or emicizumab therapies. For severe mutants, even a small increase in FVIII activity (to over 1% of WT) is sufficient to alleviate spontaneous bleeding. However, often in addition to secretion defects, severe mutations also affect FVIII cofactor functions. Although SAHA increased the secretion of most severe mutants, only two of the seven tested mutants (p.I585T and p.S603T) significantly increased the cofactor activity levels. SAHA likely increased their activities by improving the folding of these FVIII mutants. Overall, the range of activity increases suggests that SAHA could improve symptoms in patients with responsive mutations, thereby reducing their reliance on replacement therapies.

After translocation into the ER, FVIII undergoes N-glycosylation, disulfide bond formation, folding, and quality control before exiting the ER with the LMAN1-MCFD2 cargo receptor in COPII vesicles [12,13]. SAHA increased BiP and CALR levels in FVIII-expressing cells and enhanced BiP-FVIII interaction. These results suggest that SAHA enhances FVIII secretion by potentiating its interaction with BiP, which is a peptide-dependent ATPase that binds unfolded/misfolded proteins and releases them upon ATP binding [48,49]. SAHA may promote binding with ER chaperones, thereby allowing more time for FVIII to fold and increasing the amount of properly folded protein ready for trafficking out of the ER. This is consistent with a recent finding that FVIII forms amyloid that is dissolved by BiP [15]. This process would also decrease the misfolded FVIII that is degraded through the ERAD pathway [50]. The observations that the secretion and BiP interaction of individual domains of FVIII were also increased by SAHA suggest that SAHA may enhance FVIII folding on the individual domain level. Efforts have been made to identify small molecules that improve the secretion of other coagulation factors. The chemical chaperone betaine was shown to improve the folding and secretion of WT and a mutant FVIII [51]. Another compound, 4-phenylbutyrate, was found to increase a missense factor VII mutation [52] and a frequent FIX missense mutation [53].

We used two approaches to investigate the role of BiP in FVIII secretion. First, we used SubAB to decrease the intracellular BiP levels. SubAB is a serine protease bacterial toxin that enters the endoplasmic reticulum ER and inactivates BiP by cleaving off a C-terminal fragment [54]. Second, we used a specific BiP activator, BIX, to induce BiP expression through the ATF6 pathway without disturbing other ER chaperone expressions [55]. Treating cells with SubAB decreased BiP expression and FVIII secretion, whereas an inactive SubAB (with the S272A point mutation) [54] did not affect BiP expression and FVIII secretion. BIX treatment enhanced BiP expression and interaction with FVIII, mimicking the effects of SAHA treatment, suggesting that BIX also facilitates the folding and trafficking of FVIII.

Our results provide proof of the principle that PRs can provide promising treatment options for HA. Our data detail molecular mechanisms underlying the effects of PR on FVIII mutant secretion and suggest that FVIII-BiP interaction may serve as a key target for intervention by PRs. Understanding the effectiveness of PR treatments based on each patient’s mutation profile is necessary for the future development of a clinically relevant and accurate prediction model of the effectiveness of these treatments. Further studies are needed to investigate FVIII levels in animal models with missense FVIII mutations upon PR treatments. Testing the FVIII levels in the plasma of human patients treated with SAHA will further validate the role of SAHA as a PR.

## Figures and Tables

**Table 1:** List of missense mutations and their properties.

	Mutation	Codon	Protein	Domain	Exon	Patients	Severity
1	c.1834C>T	CGC-TGC	p.R612C	A2	12	242	Mild/Moderate
2	c.6506G>A	CGT-CAT	p.R2169H	C1	23	182	Moderate/Mild
3	c.6046C>T	CGG-TGG	p.R2016W	A3	19	100	Moderate/Severe
4	c.1094A>G	TAT-TGA	p.Y365C	A1	8	97	Mild
5	c.6532C>T	CGC-TGC	p.R2178C	C1	23	93	Mild
6	c.5954G>A	CGA-CAA	p.R1985Q	A3	18	89	Mild
7	c.1648C>T	CGC-TGC	p.R550C	A2	11	89	Mild/Moderate
8	c.1636C>T	CGG-TGG	p.R546W	A2	11	87	Mild
9	c.6545G>A	CGC-CAC	p.R2182H	C1	23	67	Severe/Moderate
10	c.5822A>G	AAT-AGT	p.N1941S	A3	18	67	Severe/Moderate
11	c.1649G>A	CGC-CAC	p.R550H	A2	11	64	Mild
12	c.6089G>A	AGC-AAC	p.S2030N	A3	19	63	Mild
13	c.541G>A	GTG-ATG	p.R181M	A1	4	60	Mild/Moderate
14	c.6683G>A	CGA-CAA	p.R2228Q	C2	24	57	Severe/Moderate
15	c.6744G>T	TGG-TGT	p.R2248C	C2	25	43	Mild/Moderate
16	c.902G>A	CGC-CAC	p.R301H	A1	7	43	Severe
17	c.873G>A	GAA-AAA	p.E291K	A1	7	41	Mild/Moderate
18	c.6104T>C	GTG-GCG	p.V2035A	A3	19	38	Mild
19	c.1754T>C	ATA-ACA	p.I585T	A2	12	10	Severe
20	c.1801A>G	AAC-GAC	p.N601D	A2	12	4	Severe
21	c.1808G>C	AGC-ACC	p.S603T	A2	12	1	Severe
22	c.1898T>C	ATG ACG	p.M633T	A2	12	1	Mild
23	c.1462G>A	GCA-TCA	p.A488S	A2	10	1	Not reported

Missense mutations are listed in the order of the number of patients reported in the Coagulation Factor Variant Databases.

## Data Availability

The data supporting this study are included within this article and the supplementary files. Further inquiries can be directed to the corresponding author.

## Supplementary Materials

The following supporting information can be downloaded at https://www.mdpi.com/article/10.3390/biom15040458/s1: Figure S1: High-throughput screening of 480 natural plant compounds; Figure S2: Chemical structure of suberoylanilide hydroxamic acid (SAHA); Figure S3: Effects of different concentrations of SAHA on FVIIII activity; Figure S4: The secretion time course of WT A2 and the A2-M633T mutant in cell culture; Figure S5: Effects of different concentrations of BIX along with 2.5 µM SAHA on FVIII activity; Table S1: Primary and secondary screening of 978 FDA-approved small molecules and Table S2: Primary screening of 480 F480 natural plant compounds. File S1: Original images for western blot.

## Abbreviations

The following abbreviations are used in this manuscript:

HA	hemophilia A
FVIII	factor VIII
FIX	factor IX
FL	full-length
BDD	B-domain-deleted
SAHA	suberoylanilide hydroxamic acid
EC50	half maximal effective concentration
ER	endoplasmic reticulum
BiP	immunoglobulin binding protein
CANX	calnexin
CALR	calreticulin
COPII	coat protein complex II
WT	wild type
UPR	unfolded protein response
ERAD	ER-associated degradation
PR	proteostasis regulator
FDA	Food and Drug Administration
DMSO	dimethyl sulfoxide
BIX	BiP Inducer X
FBS	Fetal Bovine Serum
DMEM	Dulbecco’s Modified Eagle’s Medium
Gluc	Gaussia luciferase
HTS	high-throughput screening
RLU	relative luciferase unit
HDACi	histone deacetylase inhibitor
