# Supplementary material for: Cell-Based Small-Molecule Screening Identifying Proteostasis Regulators Enhancing Factor VIII Missense Mutant Secretion
Source: Biomolecules. 2025 Mar 21;15(4):458. doi: 10.3390/biom15040458 (PMC12024529; doi:10.3390/biom15040458)
Supplement: Supplementary file 1 [file biomolecules-15-00458-s001.zip › biomolecules-3505096-supplementary new version/Supplementary figure S1-S5.pdf]

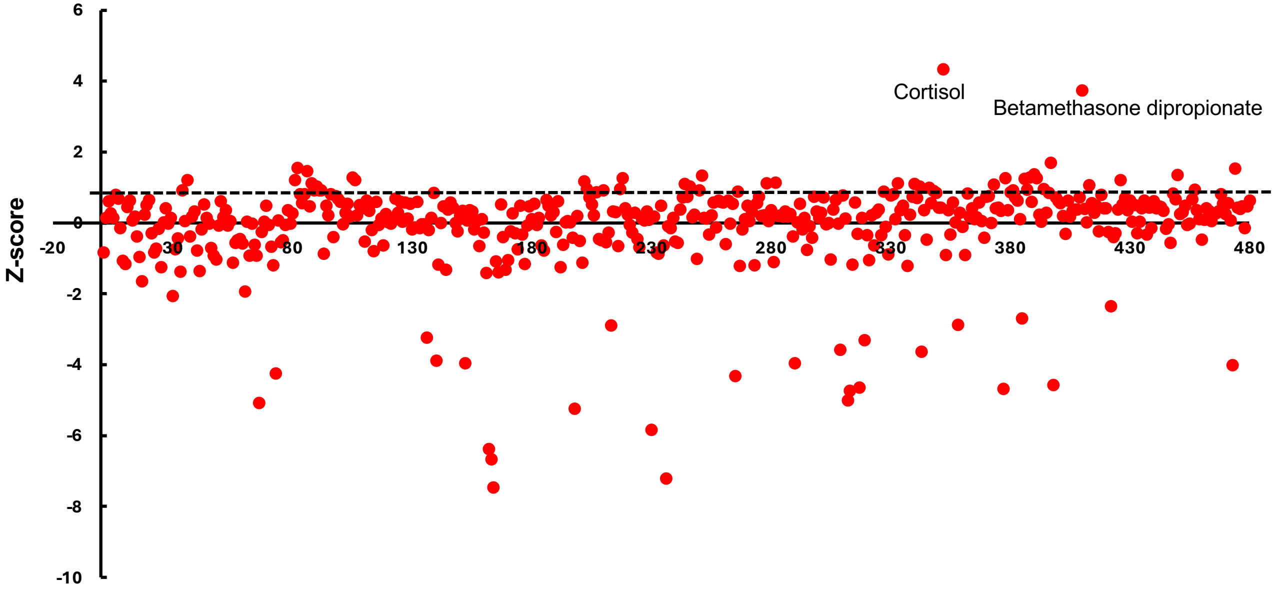

Figure S1. High-throughput screening of 480 natural plant compounds. Z-scores for each compound were calculated based on effects on p.N1941S BDD-Gluc secretion. The X-axis represents the cumulative number of compounds screened, with each dot corresponding to one compound. Cortisol and betamethasone dipropionate (another cortisol drug) were the only compounds with Z-scores ≥2.0 (3.75 and 4.35, respectively).

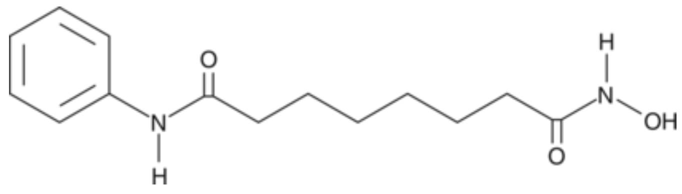

Figure S2. Chemical structure of suberoylanilide hydroxamic acid (SAHA).

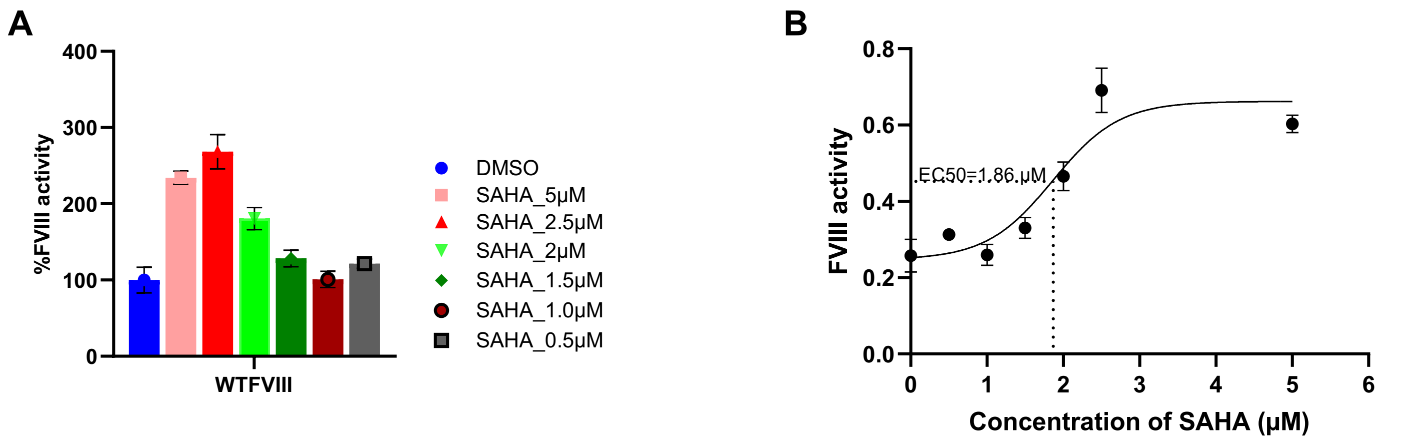

Figure S3. Effects of different concentrations of SAHA on FVIIII activity. (A) HEK293T cells were transfected with WT FL-FVIII and treated with the indicated concentrations of SAHA. FVIII activity levels in the culture media were measured and expressed as percentages of DMSO-treated cells. Data are presented as the mean ± SD from three independent experiments. (B) EC50 of SAHA was calculated based on dose dependent effects on HEK293T cells transfected with WT FL-FVIII. Graph was plotted using nonlinear regression parameters in GraphPad version 10.

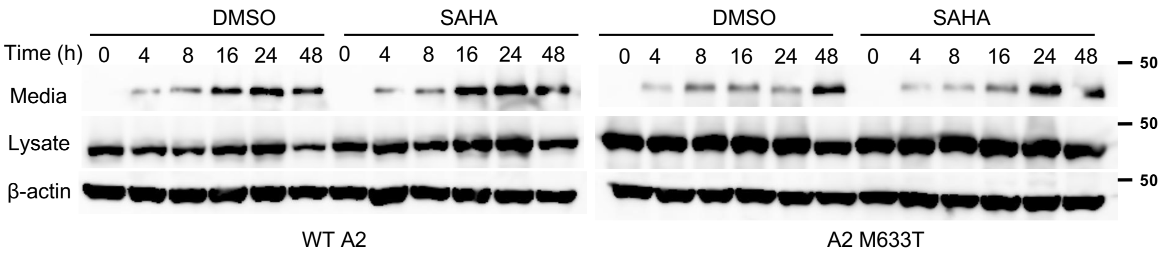

Figure S4. The secretion time course of WT A2 and the A2-M633T mutant in cell culture. HEK293 cells transfected with the WT A2 domain or A2 with the M633T mutation were treated with DMSO or SAHA (2.5 μM) at 24 h post transfection. At the indicated time after treatments, conditioned media and cell lysates were prepared and analyzed by immunoblotting with anti-FLAG or anti-β-actin antibodies.

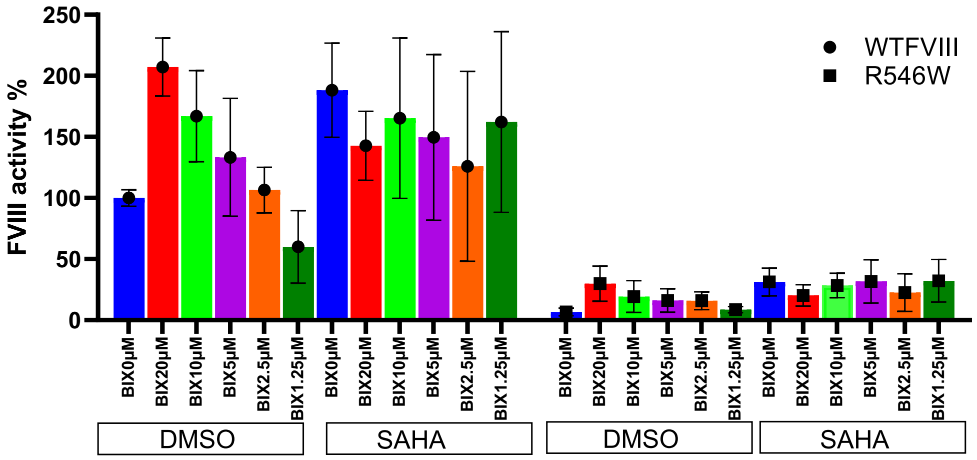

Figure S5. Effects of different concentrations of BIX along with 2.5 μM SAHA on FVIII activity. HEK293T cells transfected with WT FL-FVIII or the R546W missense mutant were treated with SAHA (2.5 μM) and the indicated concentrations of BIX for 24 h. FVIII activity levels in the culture media were measured and expressed as percentages of WT FVIII-transfected cells treated with DMSO. There were no further increases in WT-FVIII or the R546W mutant FVIII secretion, suggesting that the two compounds act on the same pathway. Data are presented as mean ± SD from three independent experiments.
