# Supplementary figures and images for: Cell-Based Small-Molecule Screening Identifying Proteostasis Regulators Enhancing Factor VIII Missense Mutant Secretion
Source: Biomolecules. 2025 Mar 21;15(4):458. doi: 10.3390/biom15040458 (PMC12024529; doi:10.3390/biom15040458)

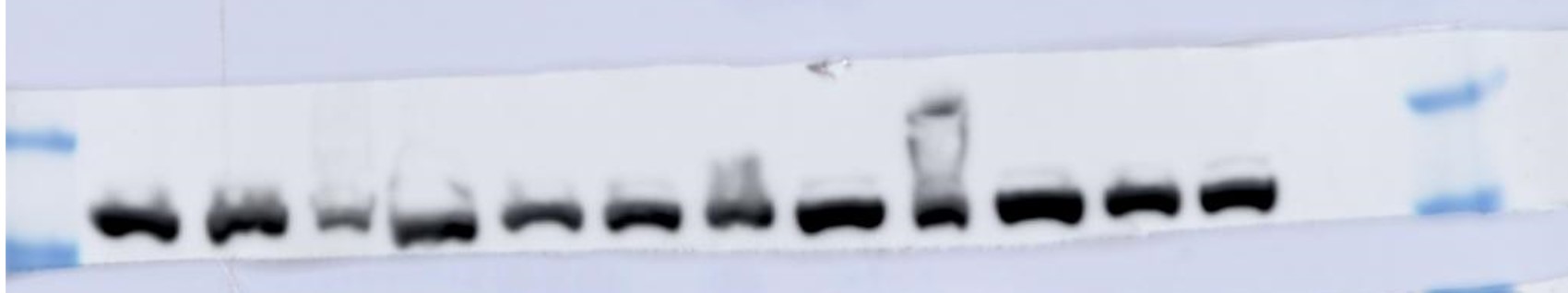

Supplement: Supplementary file 1 [file biomolecules-15-00458-s001.zip › biomolecules-3505096-supplementary new version/File S1/Fig5-A1.jpg]

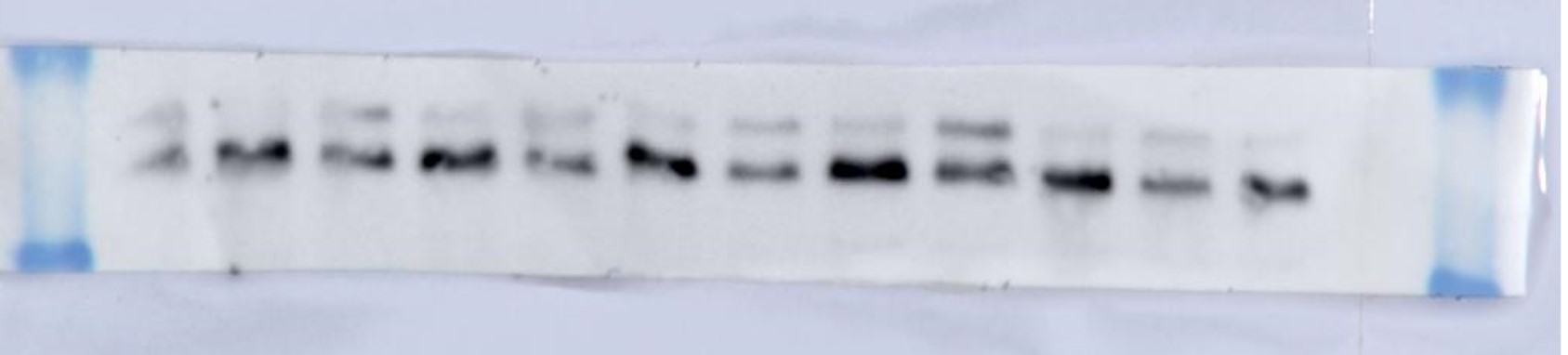

Supplement: Supplementary file 1 [file biomolecules-15-00458-s001.zip › biomolecules-3505096-supplementary new version/File S1/Fig5-A2.jpg]

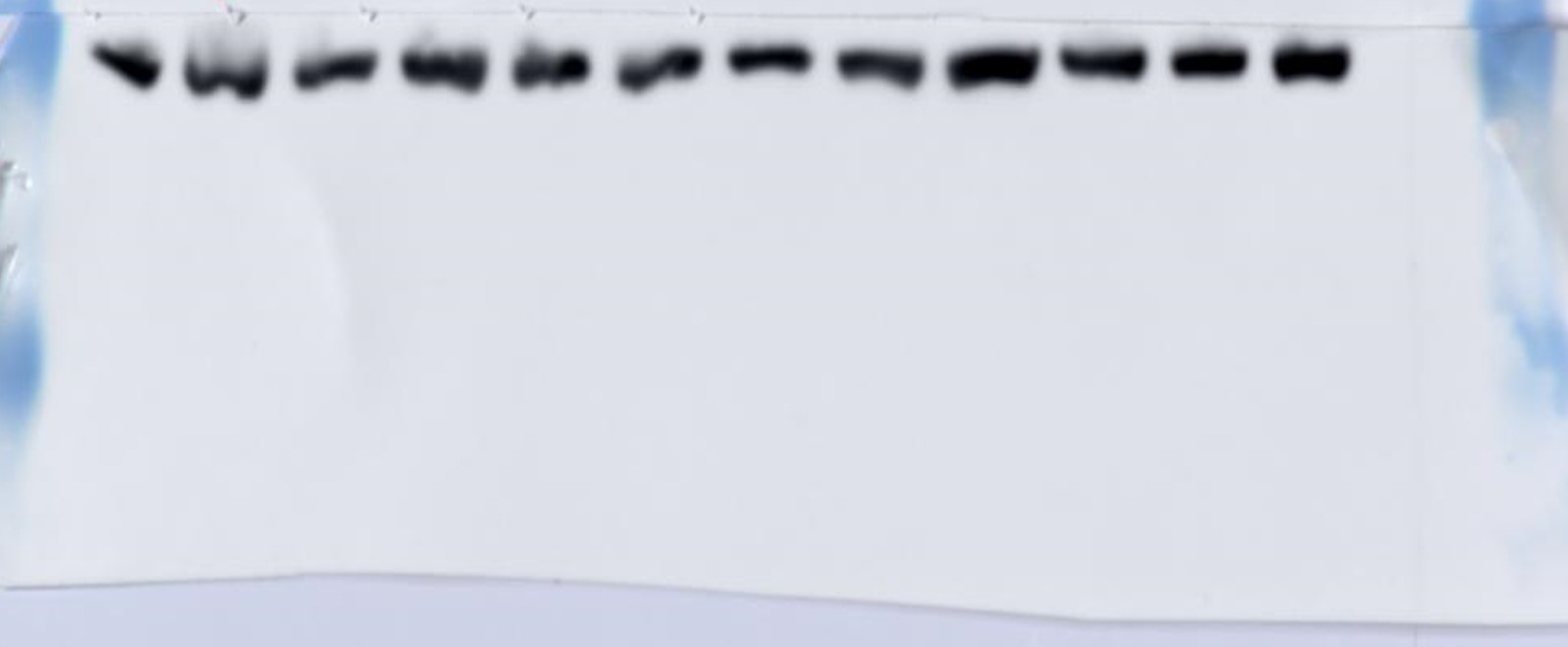

Supplement: Supplementary file 1 [file biomolecules-15-00458-s001.zip › biomolecules-3505096-supplementary new version/File S1/Fig5-A3.jpg]

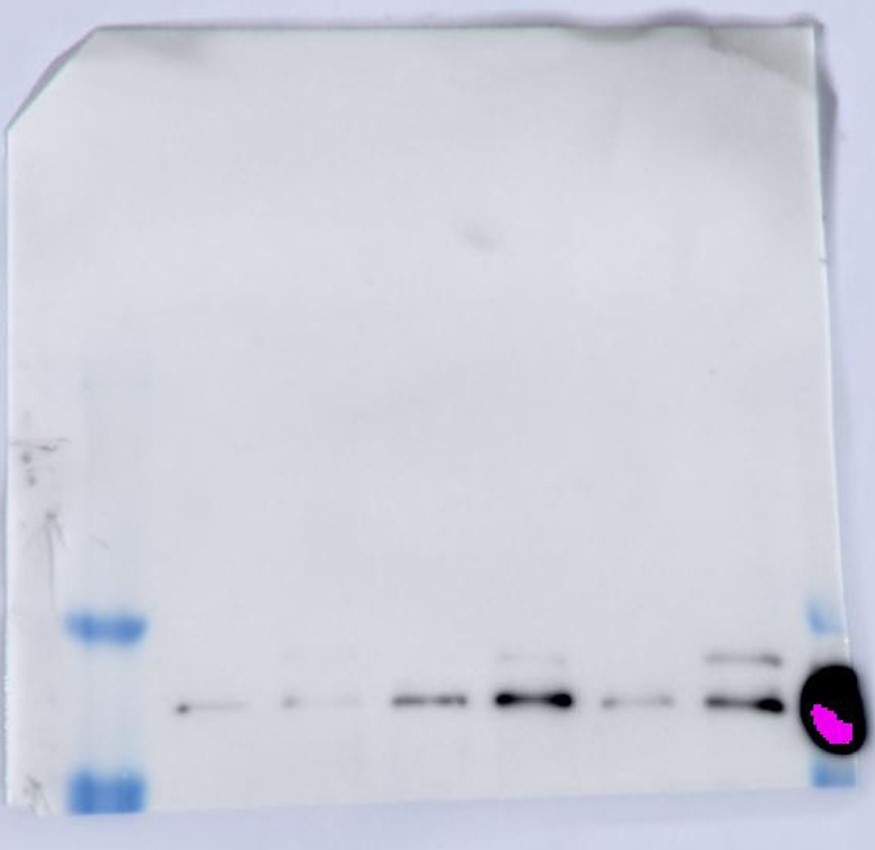

Supplement: Supplementary file 1 [file biomolecules-15-00458-s001.zip › biomolecules-3505096-supplementary new version/File S1/Fig5-B1.jpg]

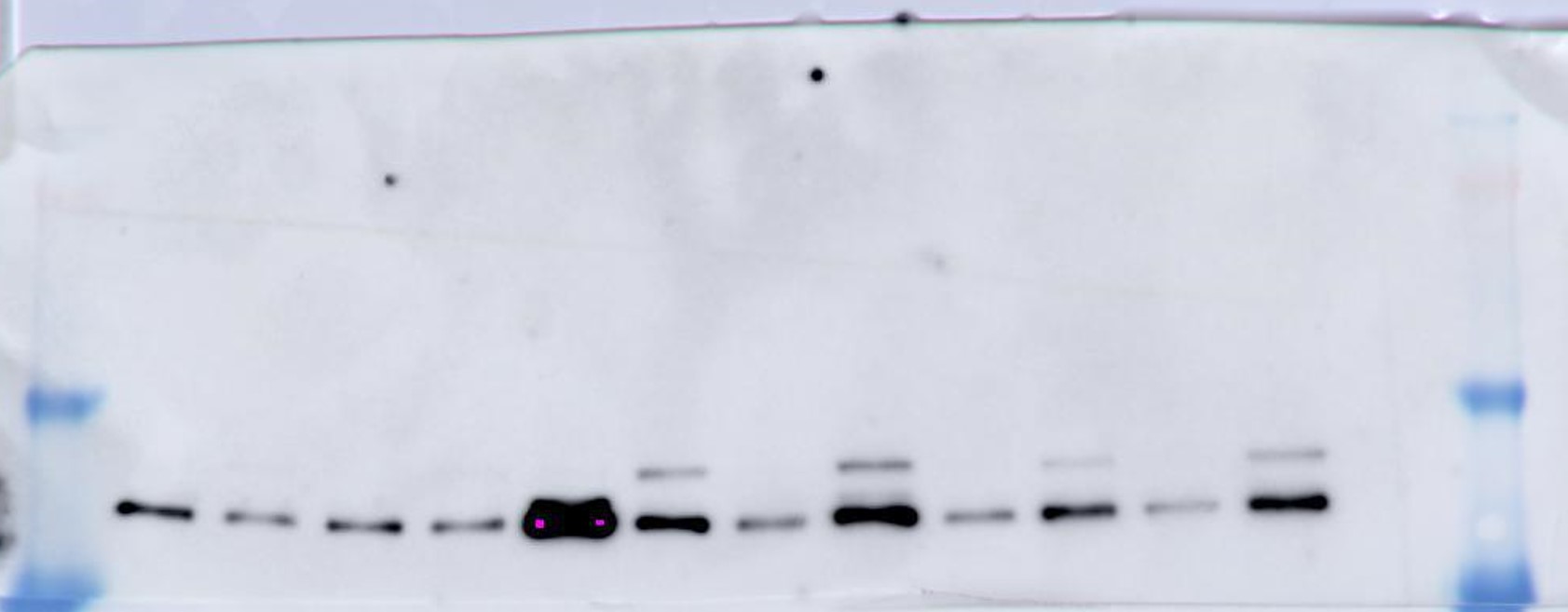

Supplement: Supplementary file 1 [file biomolecules-15-00458-s001.zip › biomolecules-3505096-supplementary new version/File S1/Fig5-B2.jpg]

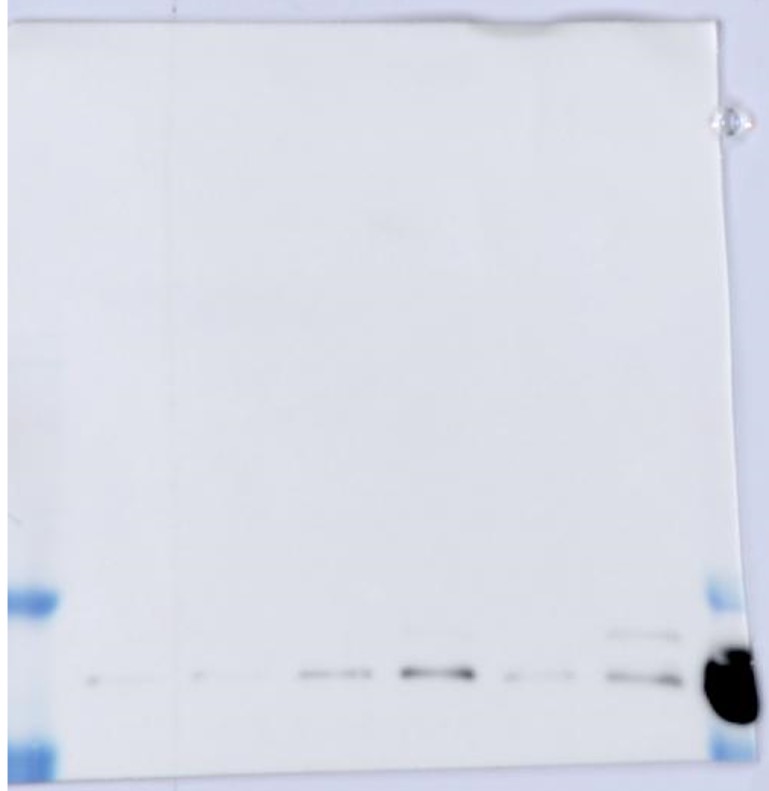

Supplement: Supplementary file 1 [file biomolecules-15-00458-s001.zip › biomolecules-3505096-supplementary new version/File S1/Fig5-B3.jpg]

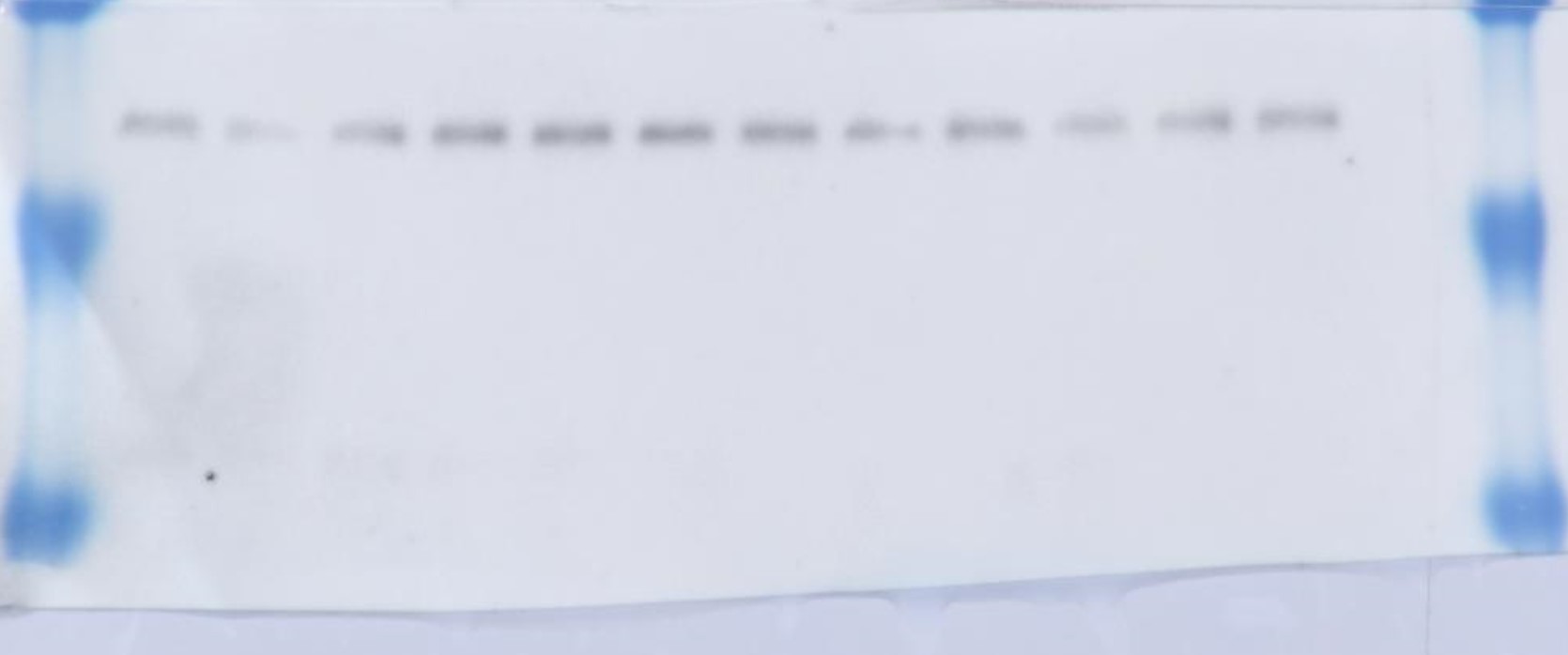

Supplement: Supplementary file 1 [file biomolecules-15-00458-s001.zip › biomolecules-3505096-supplementary new version/File S1/Fig5-B4.jpg]

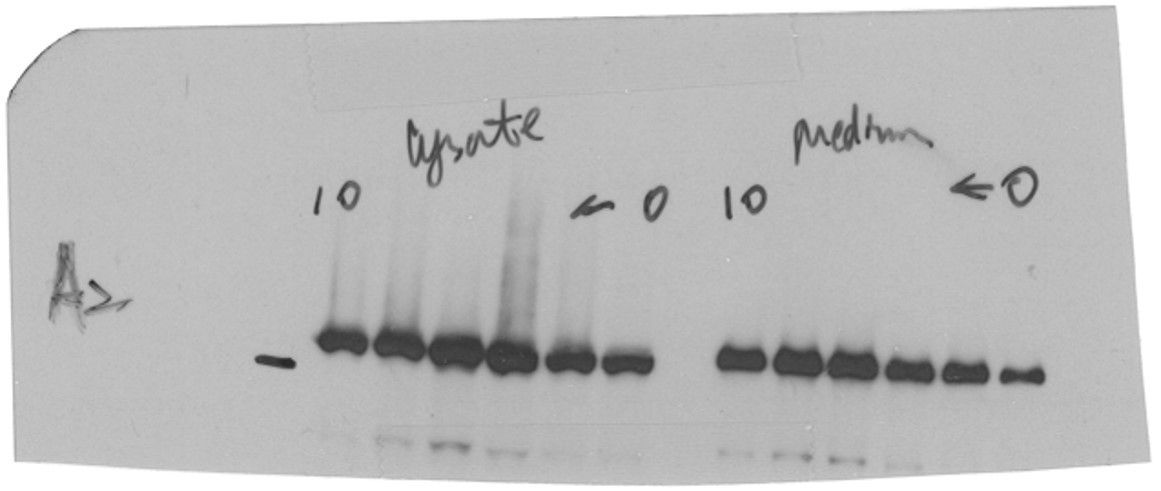

Supplement: Supplementary file 1 [file biomolecules-15-00458-s001.zip › biomolecules-3505096-supplementary new version/File S1/Fig6-1.jpg]

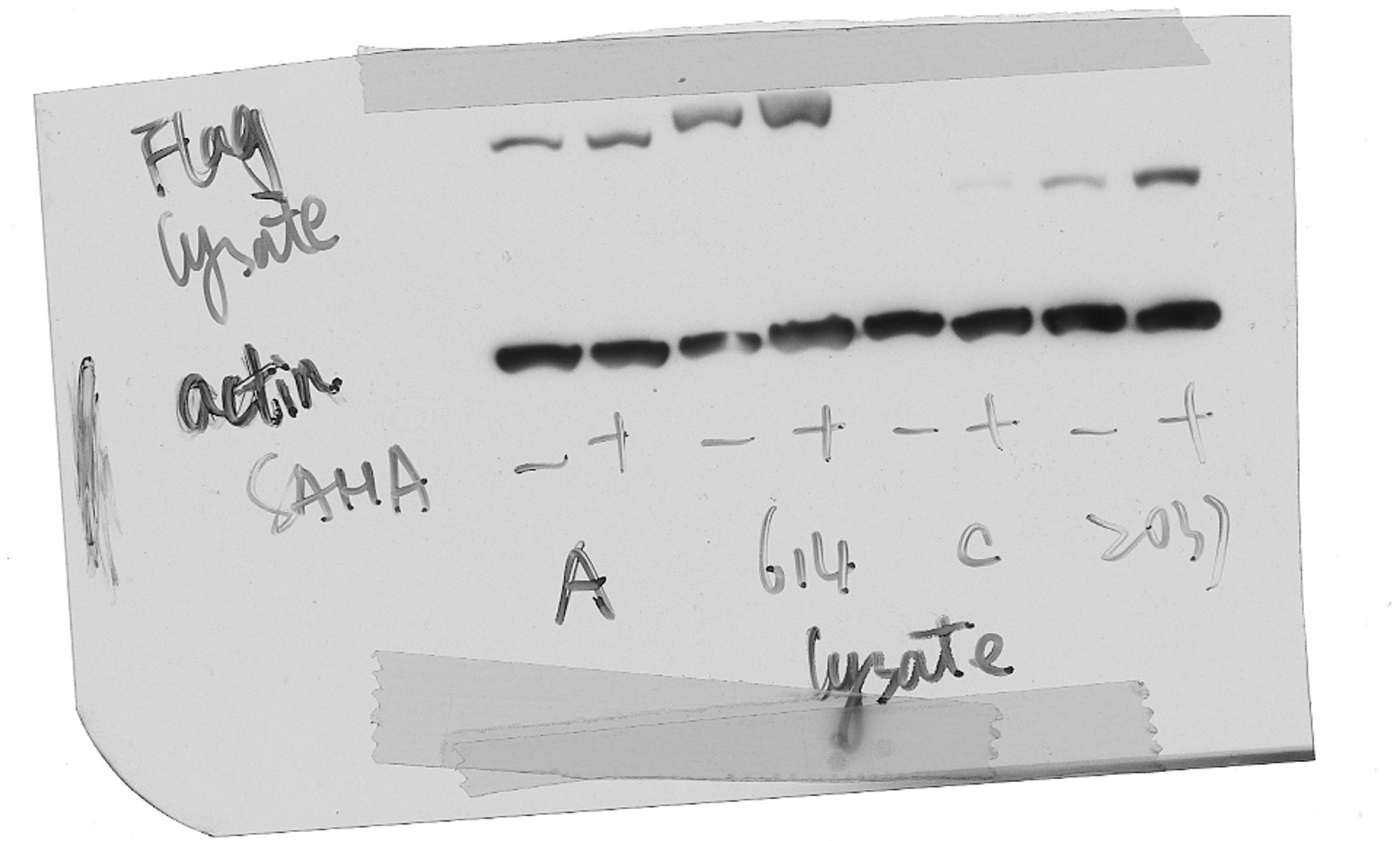

Supplement: Supplementary file 1 [file biomolecules-15-00458-s001.zip › biomolecules-3505096-supplementary new version/File S1/Fig6-10.jpg]

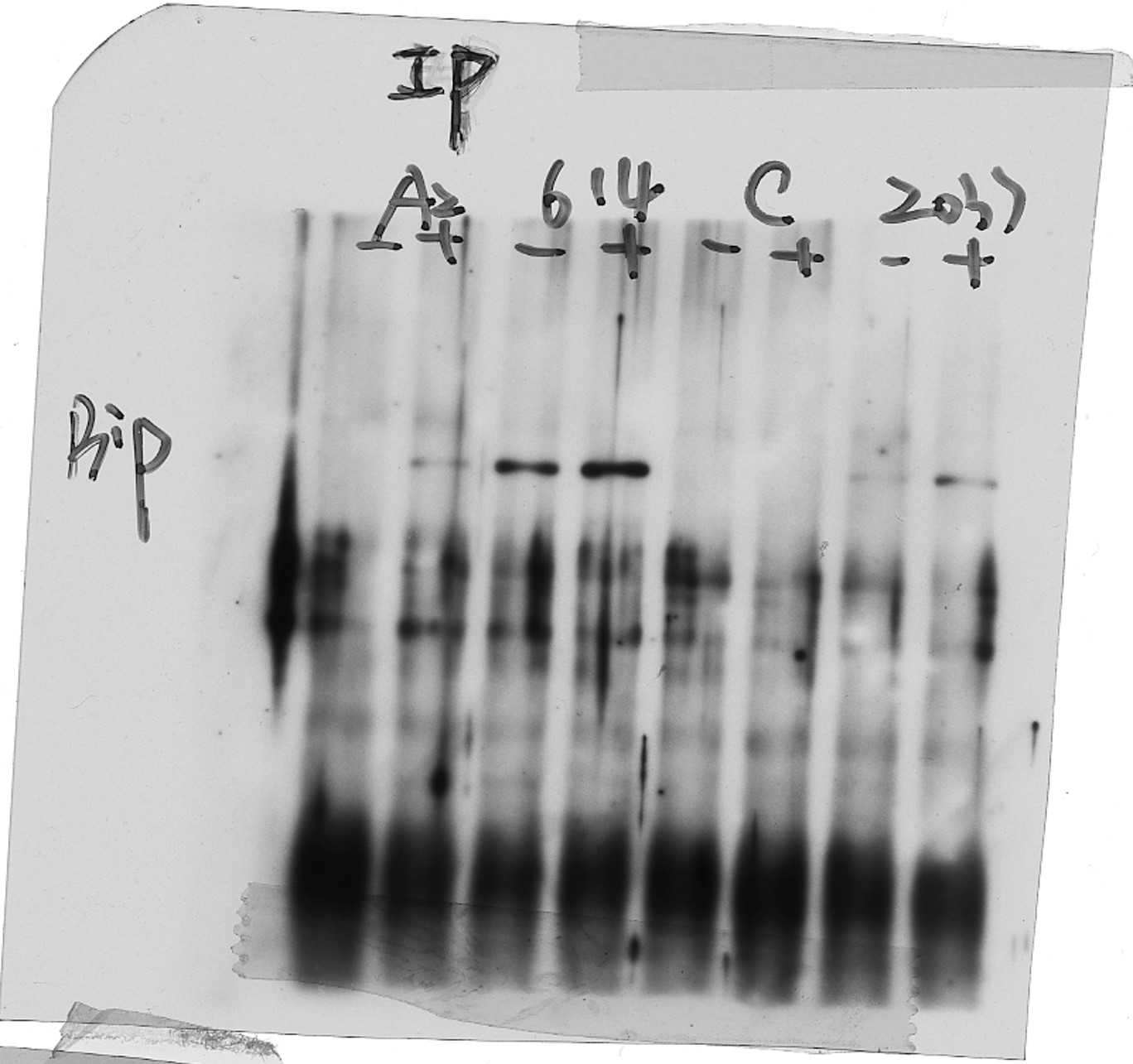

Supplement: Supplementary file 1 [file biomolecules-15-00458-s001.zip › biomolecules-3505096-supplementary new version/File S1/Fig6-11.jpg]

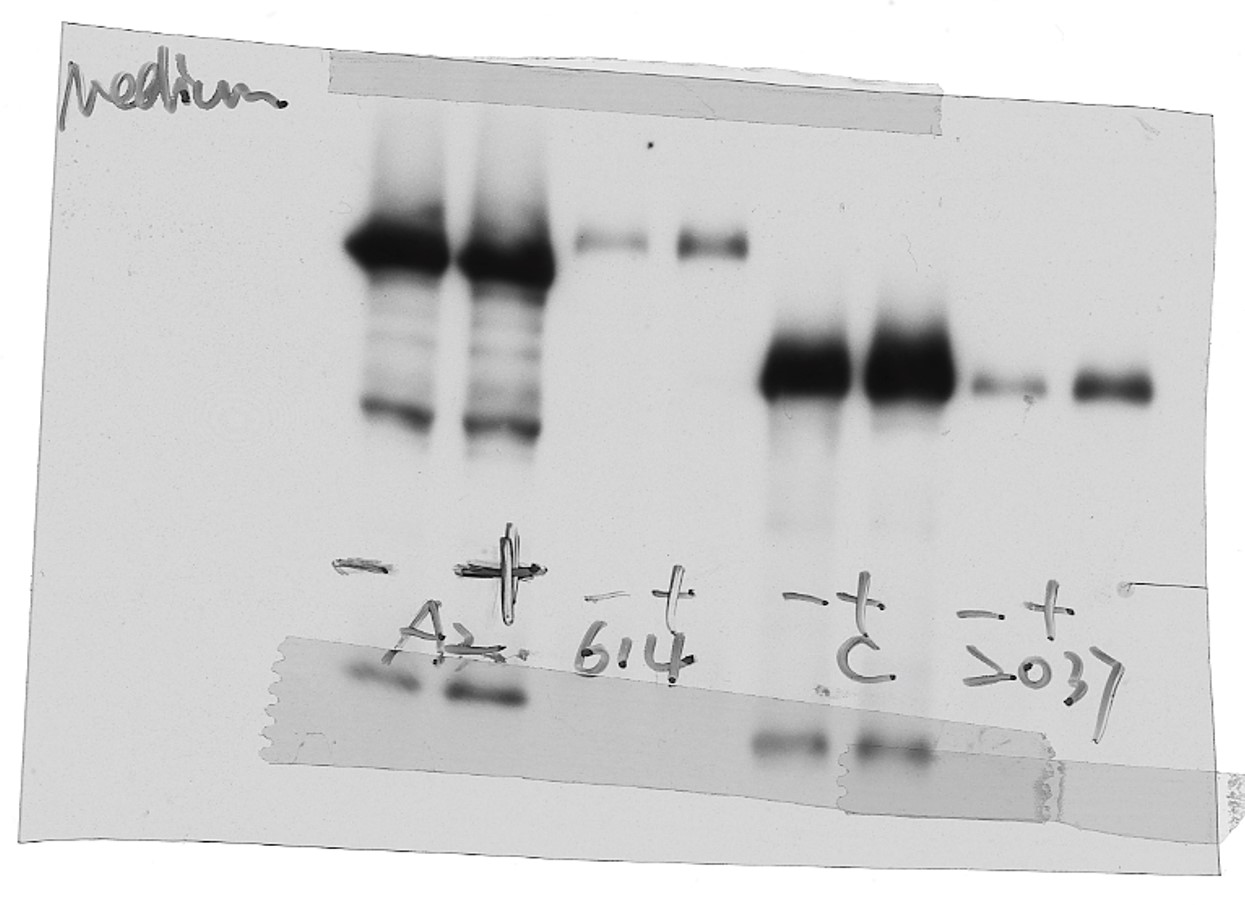

Supplement: Supplementary file 1 [file biomolecules-15-00458-s001.zip › biomolecules-3505096-supplementary new version/File S1/Fig6-12.jpg]

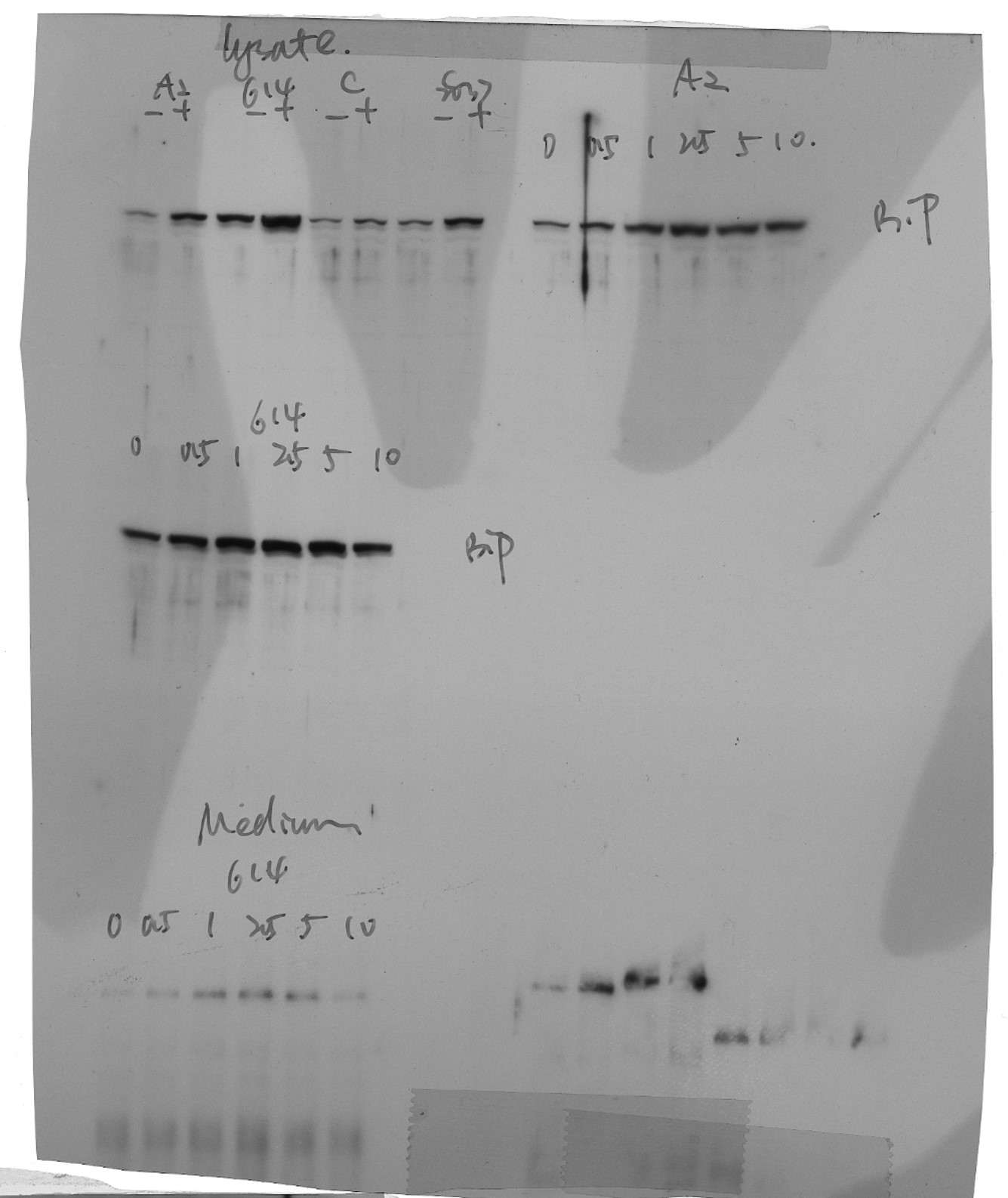

Supplement: Supplementary file 1 [file biomolecules-15-00458-s001.zip › biomolecules-3505096-supplementary new version/File S1/Fig6-2.jpg]

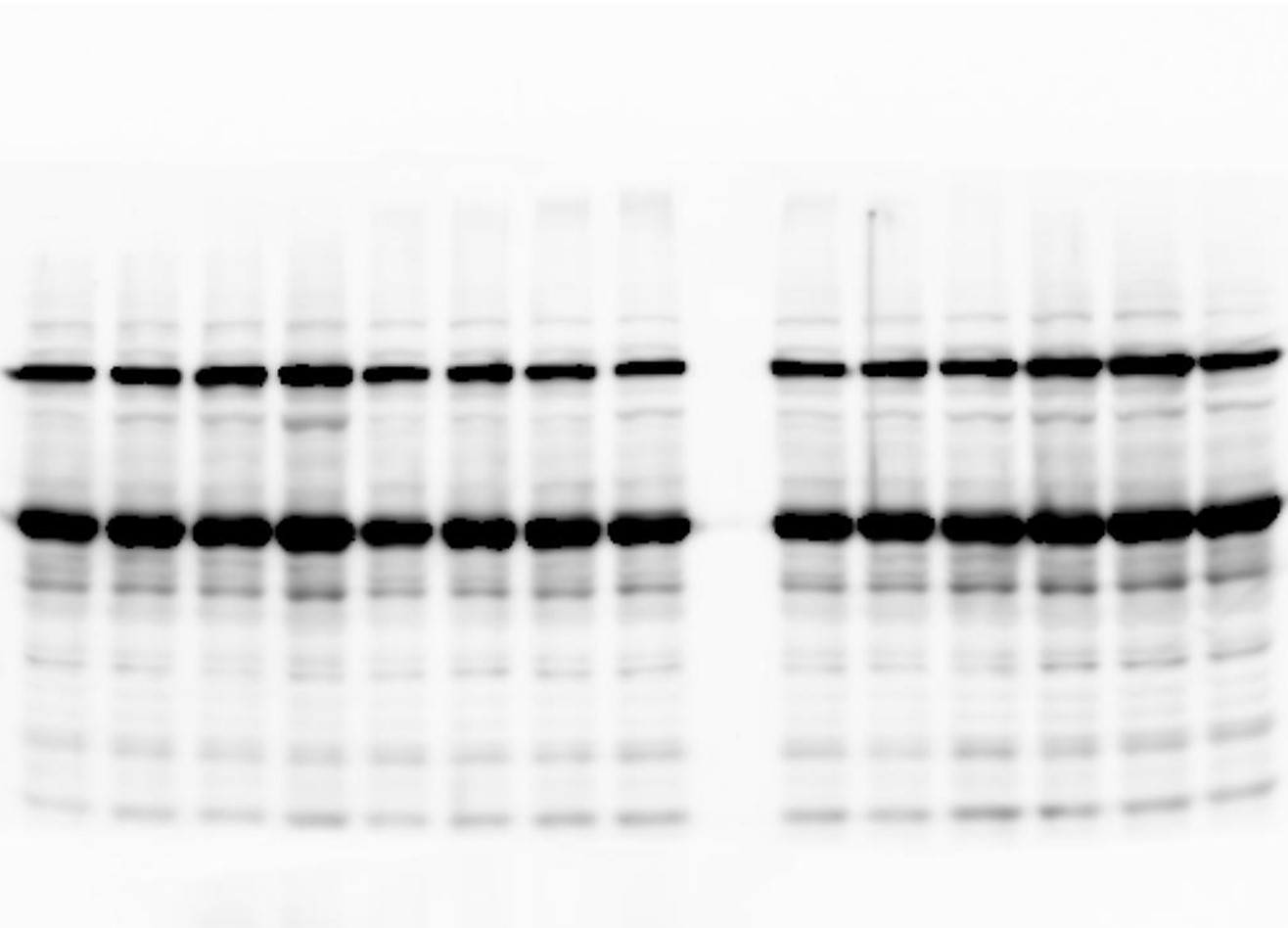

Supplement: Supplementary file 1 [file biomolecules-15-00458-s001.zip › biomolecules-3505096-supplementary new version/File S1/Fig6-3.jpg]

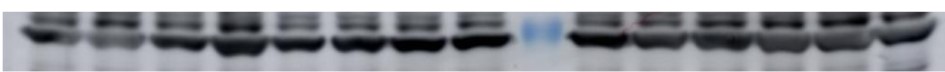

Supplement: Supplementary file 1 [file biomolecules-15-00458-s001.zip › biomolecules-3505096-supplementary new version/File S1/Fig6-4.jpg]

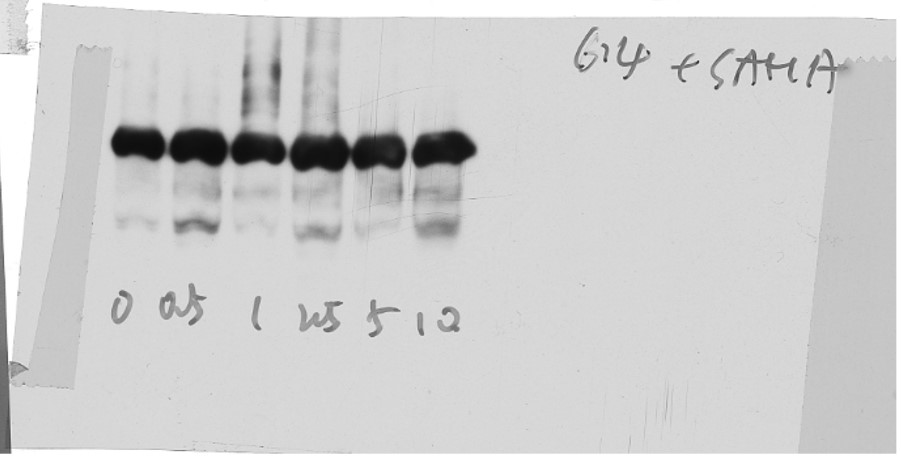

Supplement: Supplementary file 1 [file biomolecules-15-00458-s001.zip › biomolecules-3505096-supplementary new version/File S1/Fig6-5.jpg]

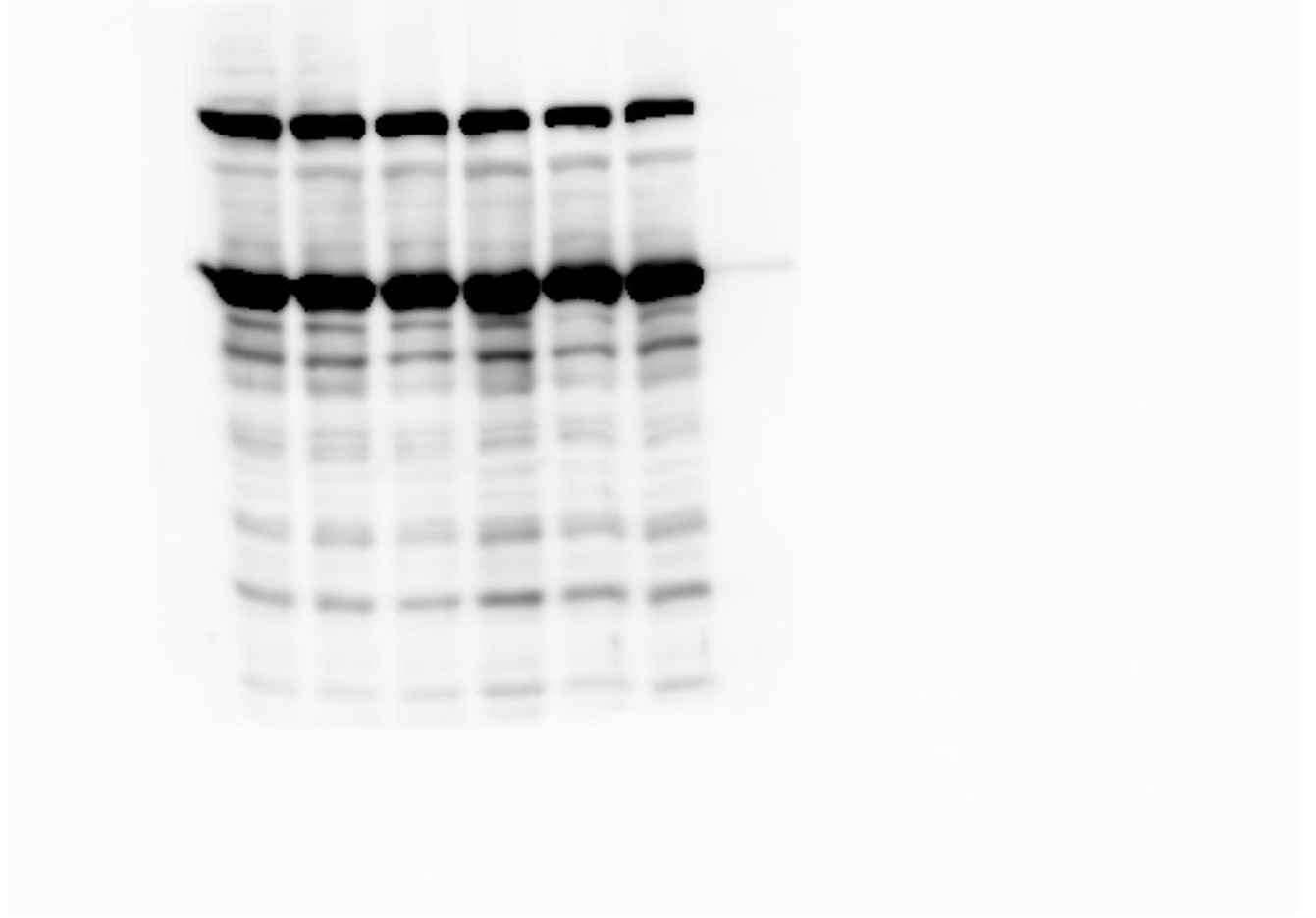

Supplement: Supplementary file 1 [file biomolecules-15-00458-s001.zip › biomolecules-3505096-supplementary new version/File S1/Fig6-6.jpg]

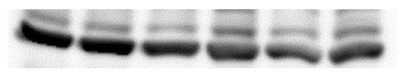

Supplement: Supplementary file 1 [file biomolecules-15-00458-s001.zip › biomolecules-3505096-supplementary new version/File S1/Fig6-7.jpg]

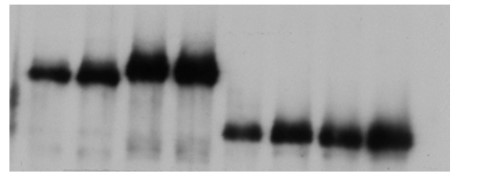

Supplement: Supplementary file 1 [file biomolecules-15-00458-s001.zip › biomolecules-3505096-supplementary new version/File S1/Fig6-8.jpg]

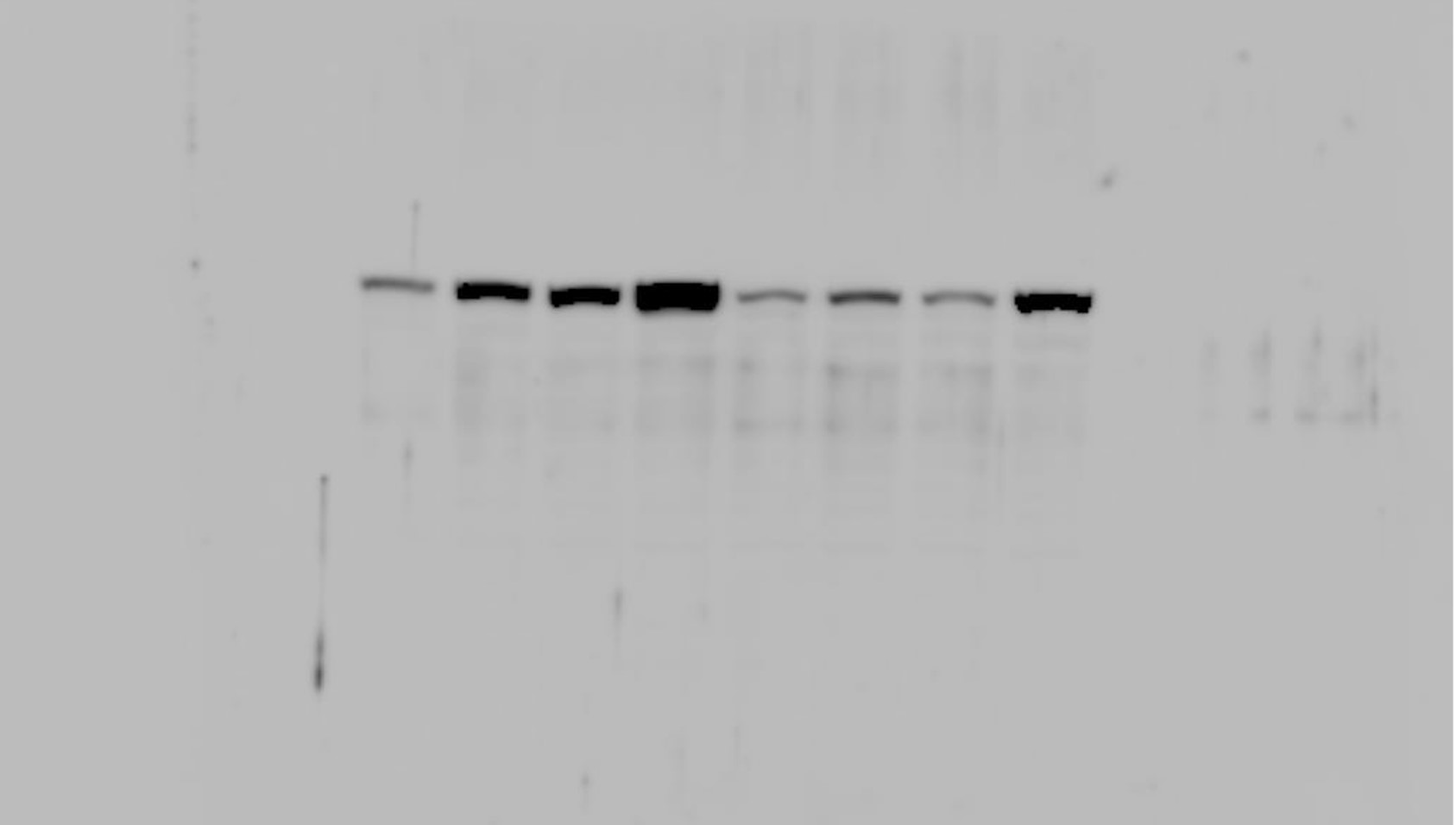

Supplement: Supplementary file 1 [file biomolecules-15-00458-s001.zip › biomolecules-3505096-supplementary new version/File S1/Fig6-9.jpg]

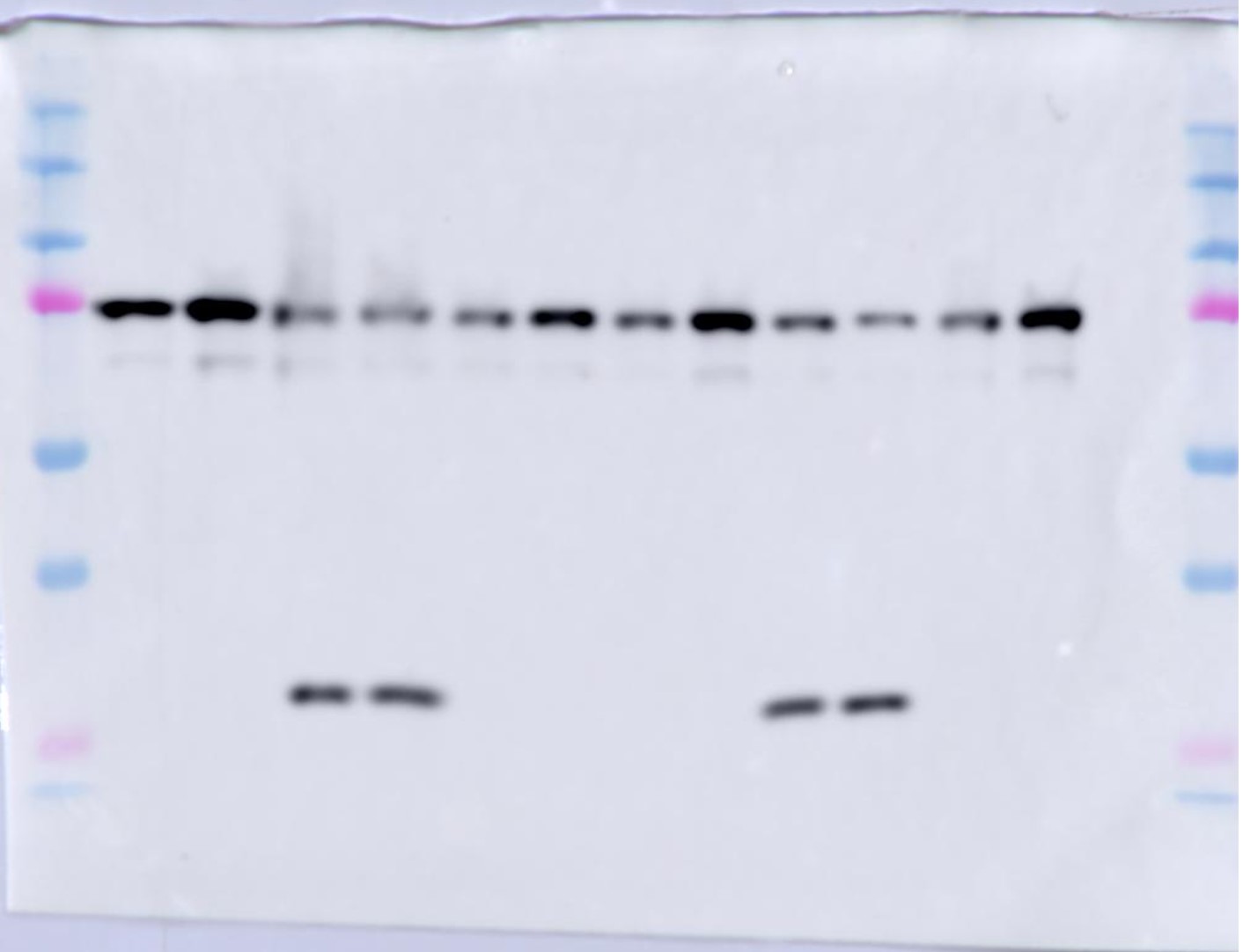

Supplement: Supplementary file 1 [file biomolecules-15-00458-s001.zip › biomolecules-3505096-supplementary new version/File S1/Fig7-A1.jpg]

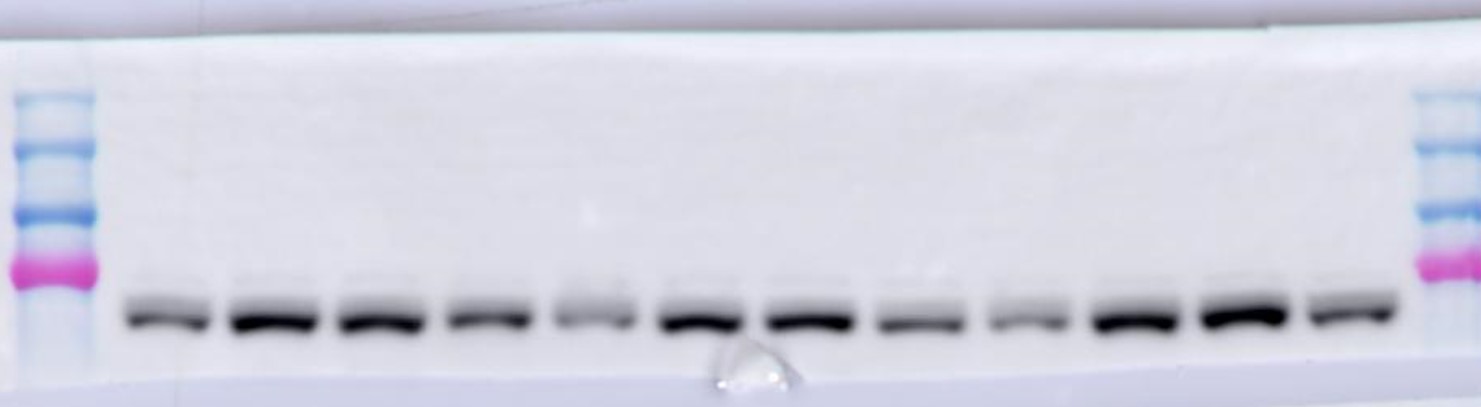

Supplement: Supplementary file 1 [file biomolecules-15-00458-s001.zip › biomolecules-3505096-supplementary new version/File S1/Fig7-C1.jpg]

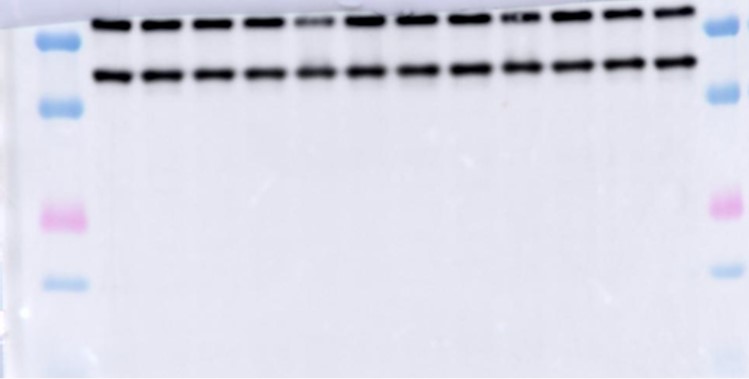

Supplement: Supplementary file 1 [file biomolecules-15-00458-s001.zip › biomolecules-3505096-supplementary new version/File S1/Fig7-C2.jpg]

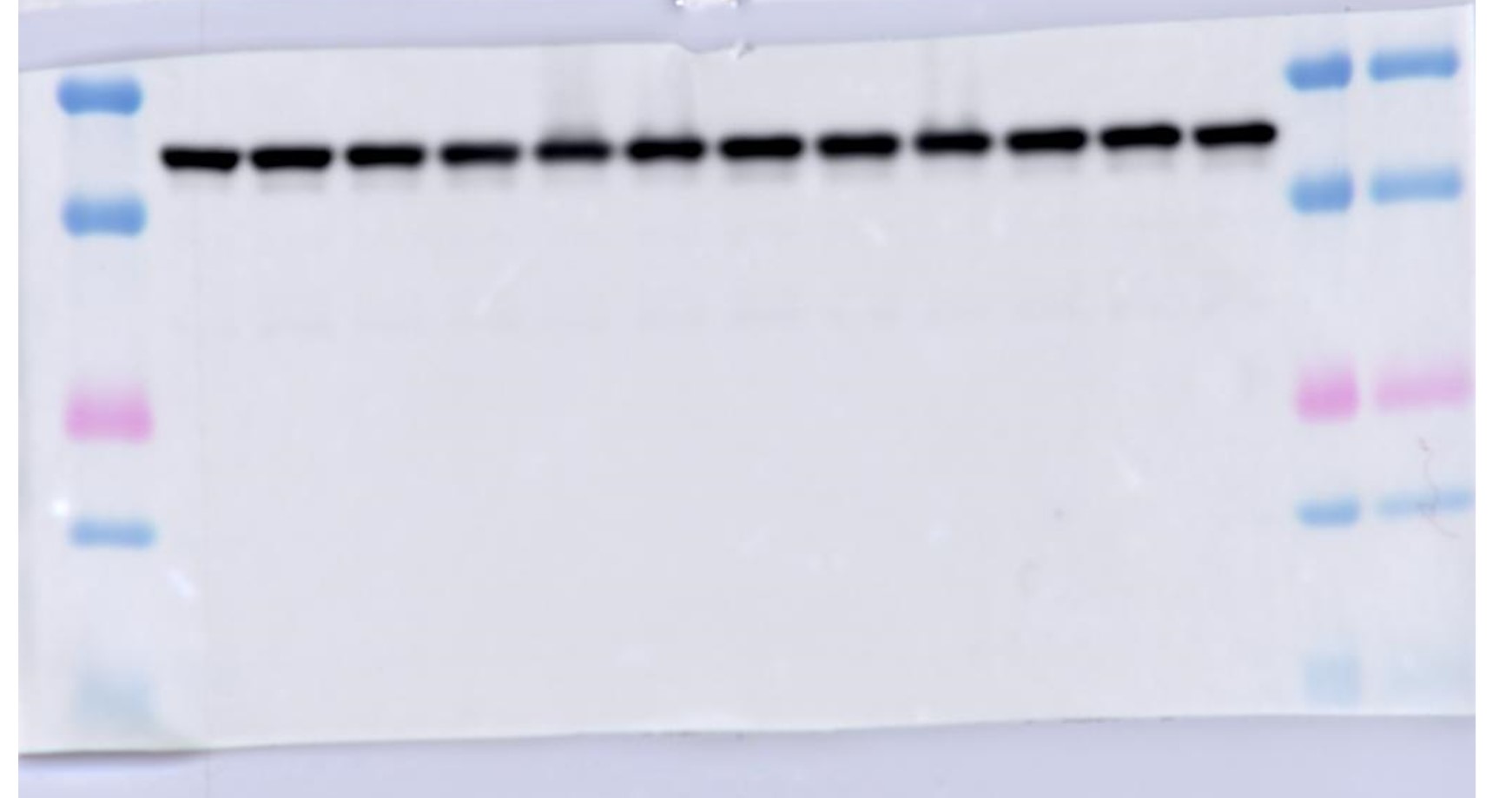

Supplement: Supplementary file 1 [file biomolecules-15-00458-s001.zip › biomolecules-3505096-supplementary new version/File S1/Fig7-C3.jpg]

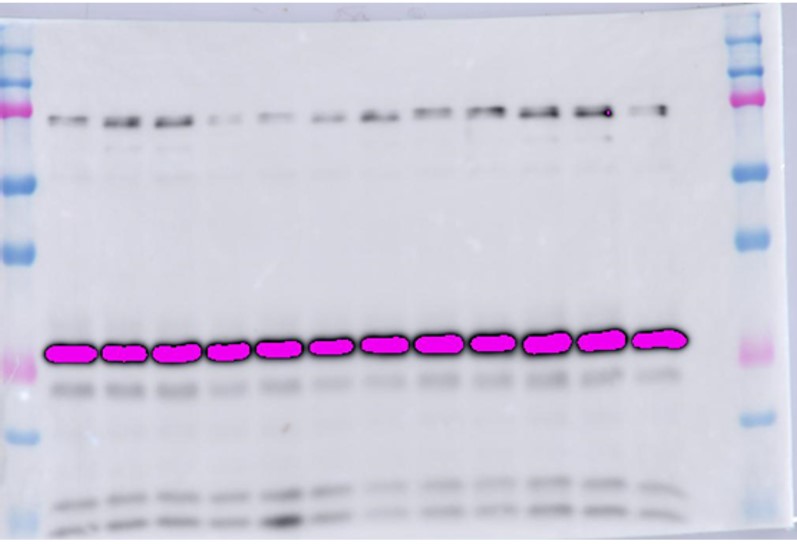

Supplement: Supplementary file 1 [file biomolecules-15-00458-s001.zip › biomolecules-3505096-supplementary new version/File S1/Fig7-C4.jpg]

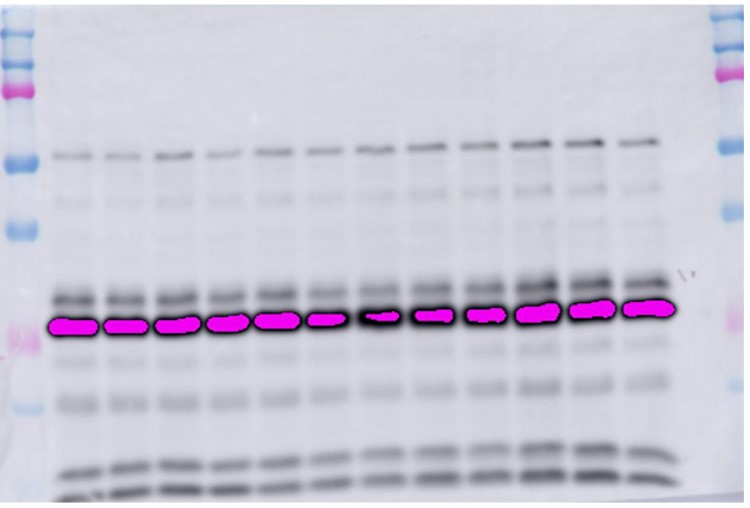

Supplement: Supplementary file 1 [file biomolecules-15-00458-s001.zip › biomolecules-3505096-supplementary new version/File S1/Fig7-C5.jpg]

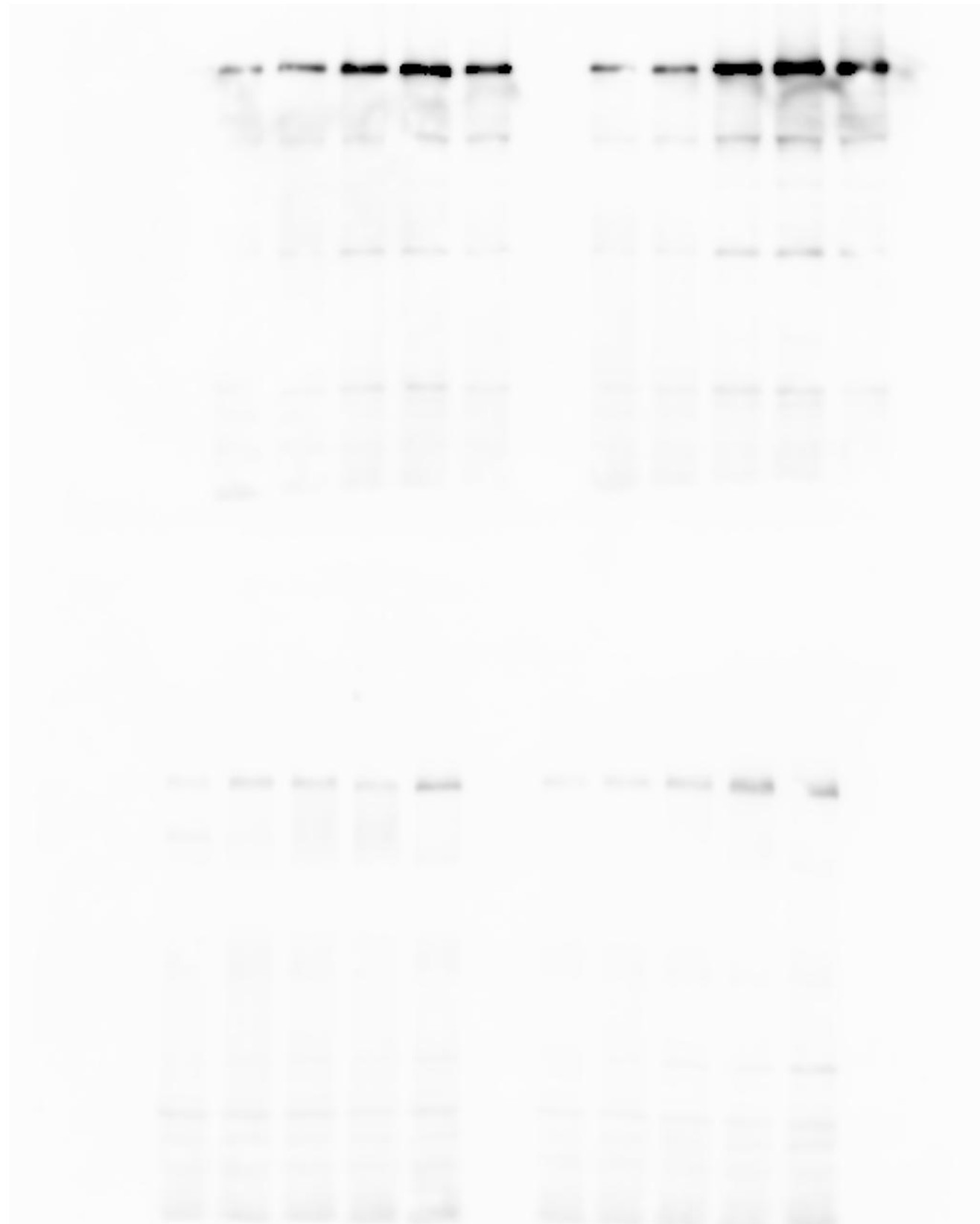

Supplement: Supplementary file 1 [file biomolecules-15-00458-s001.zip › biomolecules-3505096-supplementary new version/File S1/FigS1-1.jpg]

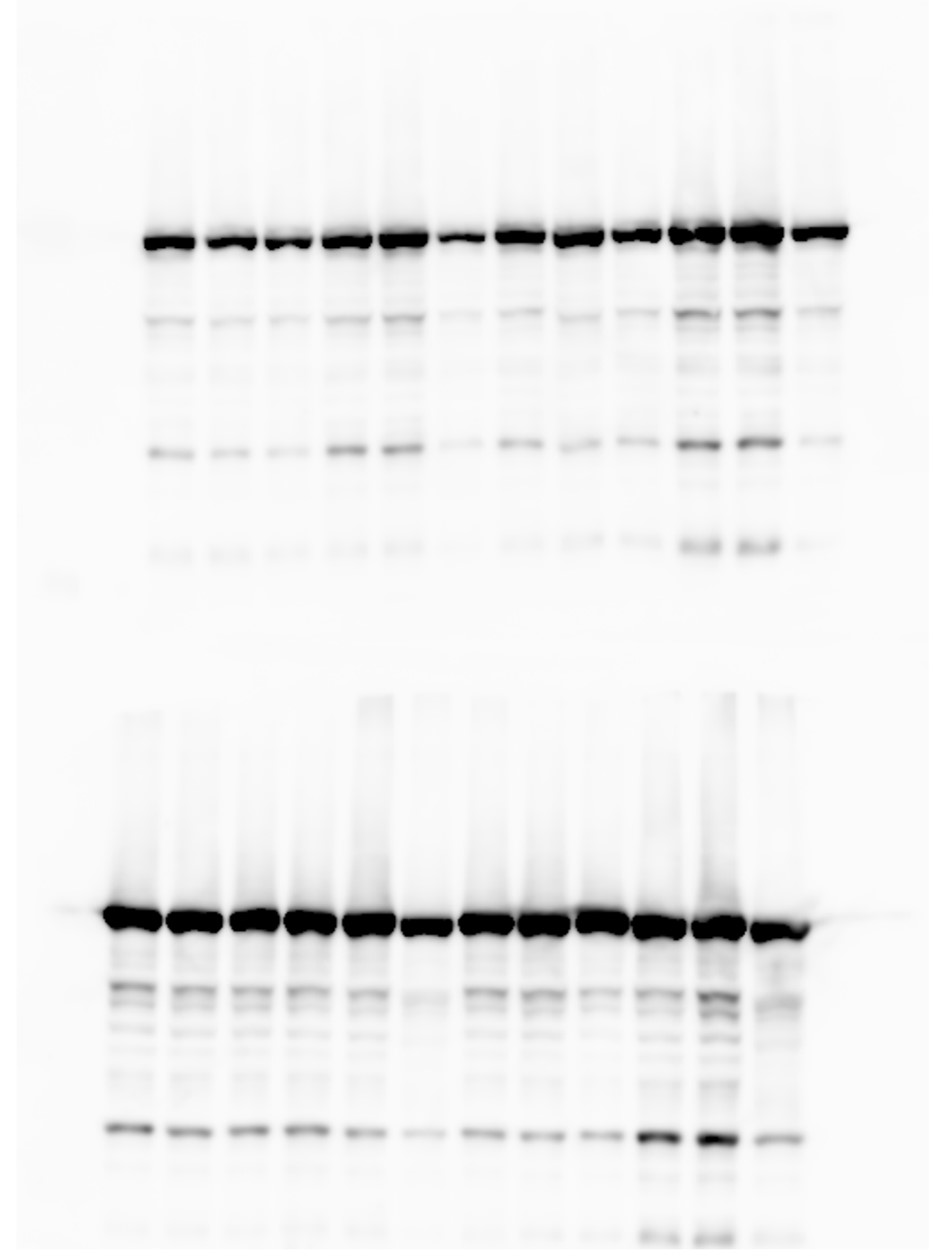

Supplement: Supplementary file 1 [file biomolecules-15-00458-s001.zip › biomolecules-3505096-supplementary new version/File S1/FigS1-2.jpg]

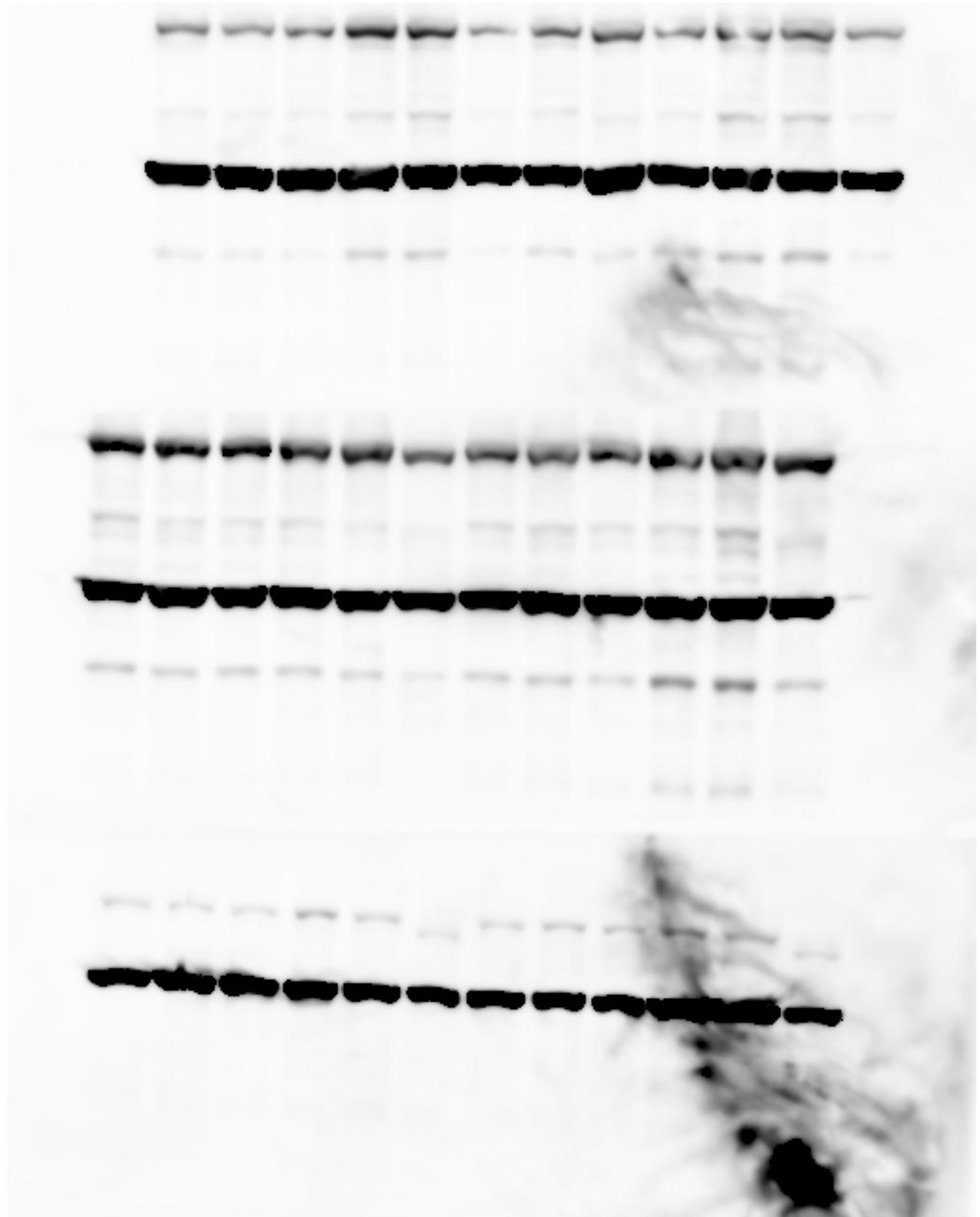

Supplement: Supplementary file 1 [file biomolecules-15-00458-s001.zip › biomolecules-3505096-supplementary new version/File S1/FigS1-3.jpg]

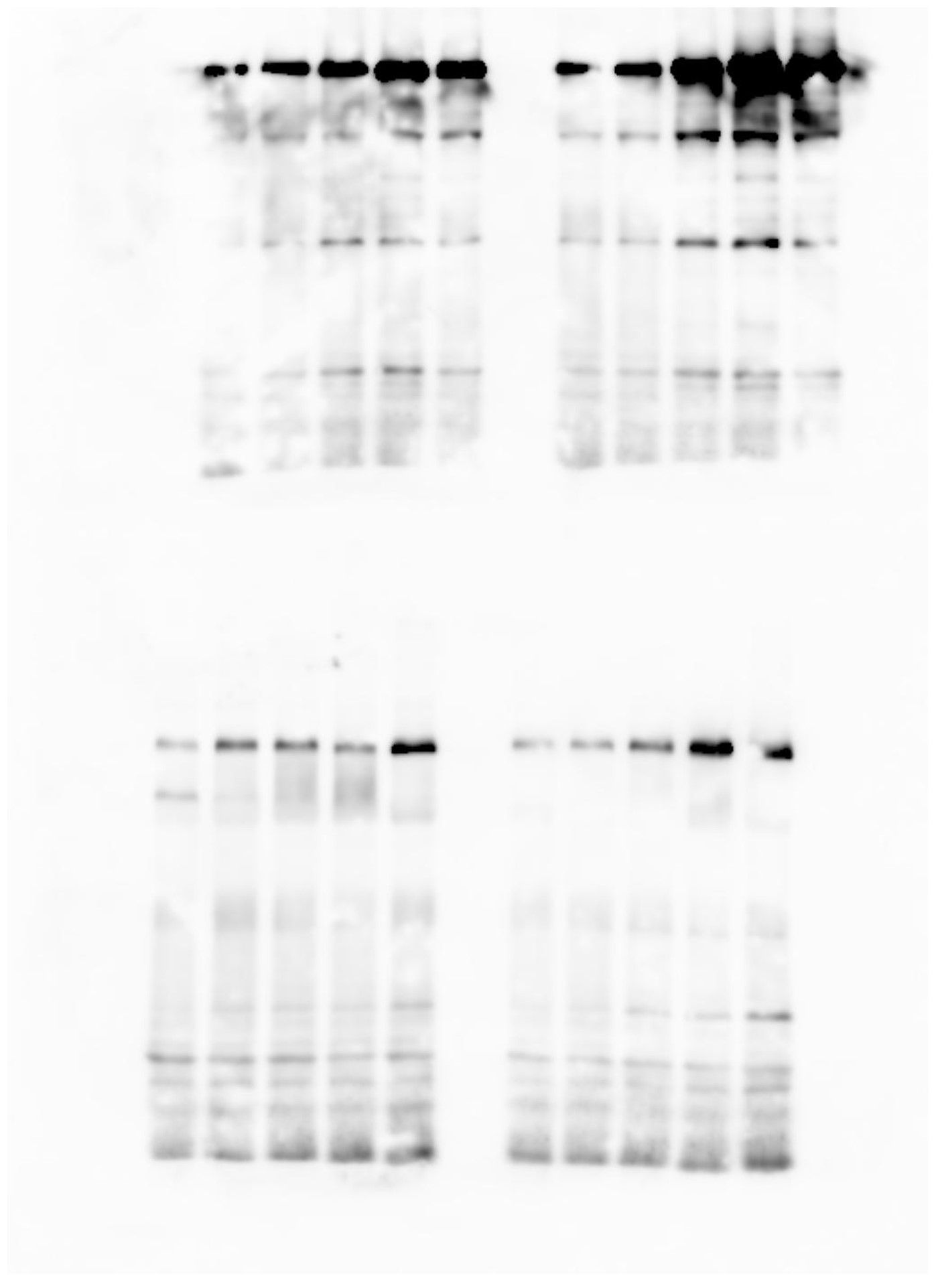

Supplement: Supplementary file 1 [file biomolecules-15-00458-s001.zip › biomolecules-3505096-supplementary new version/File S1/FigS1-4.jpg]

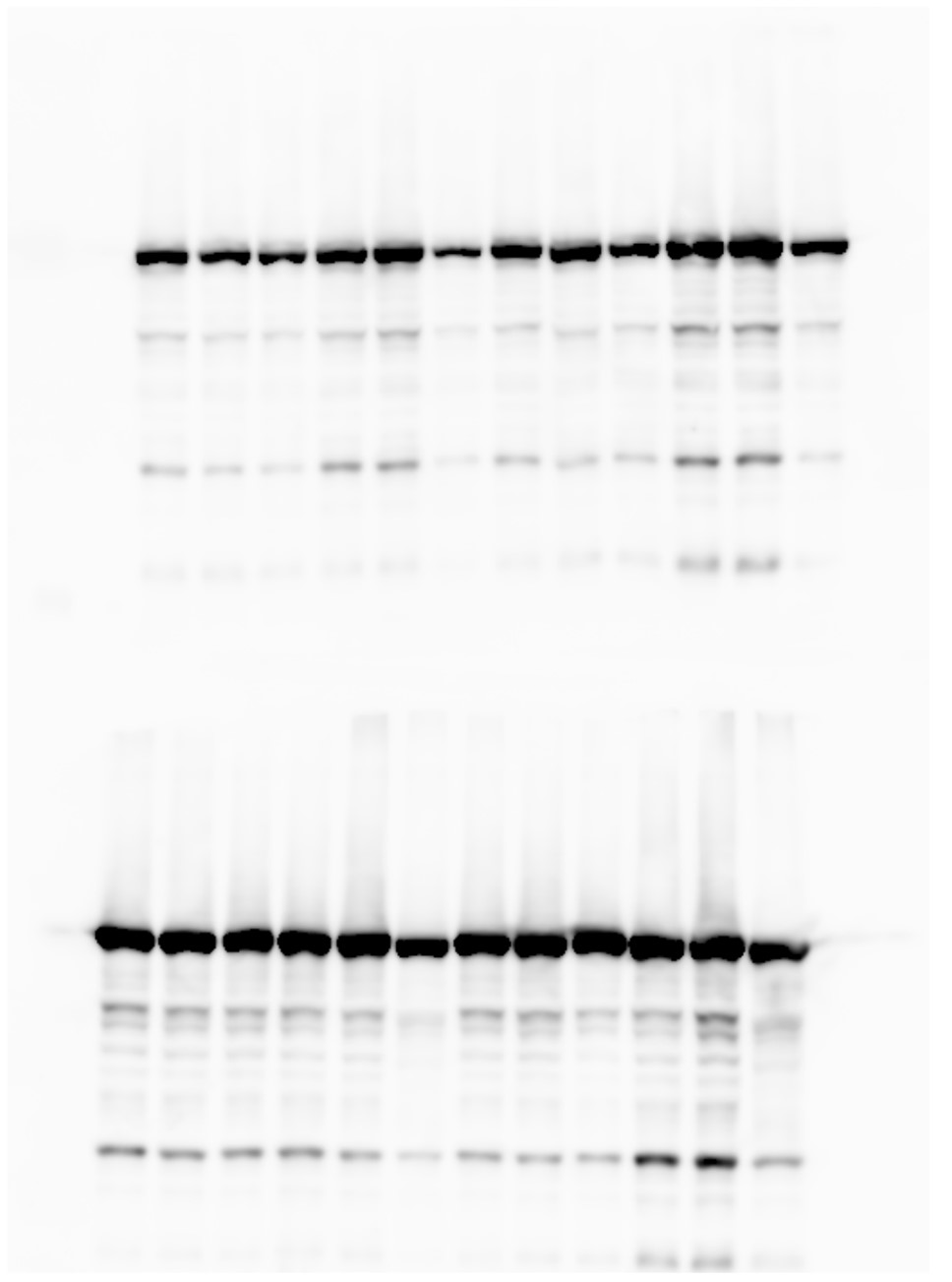

Supplement: Supplementary file 1 [file biomolecules-15-00458-s001.zip › biomolecules-3505096-supplementary new version/File S1/FigS1-5.jpg]

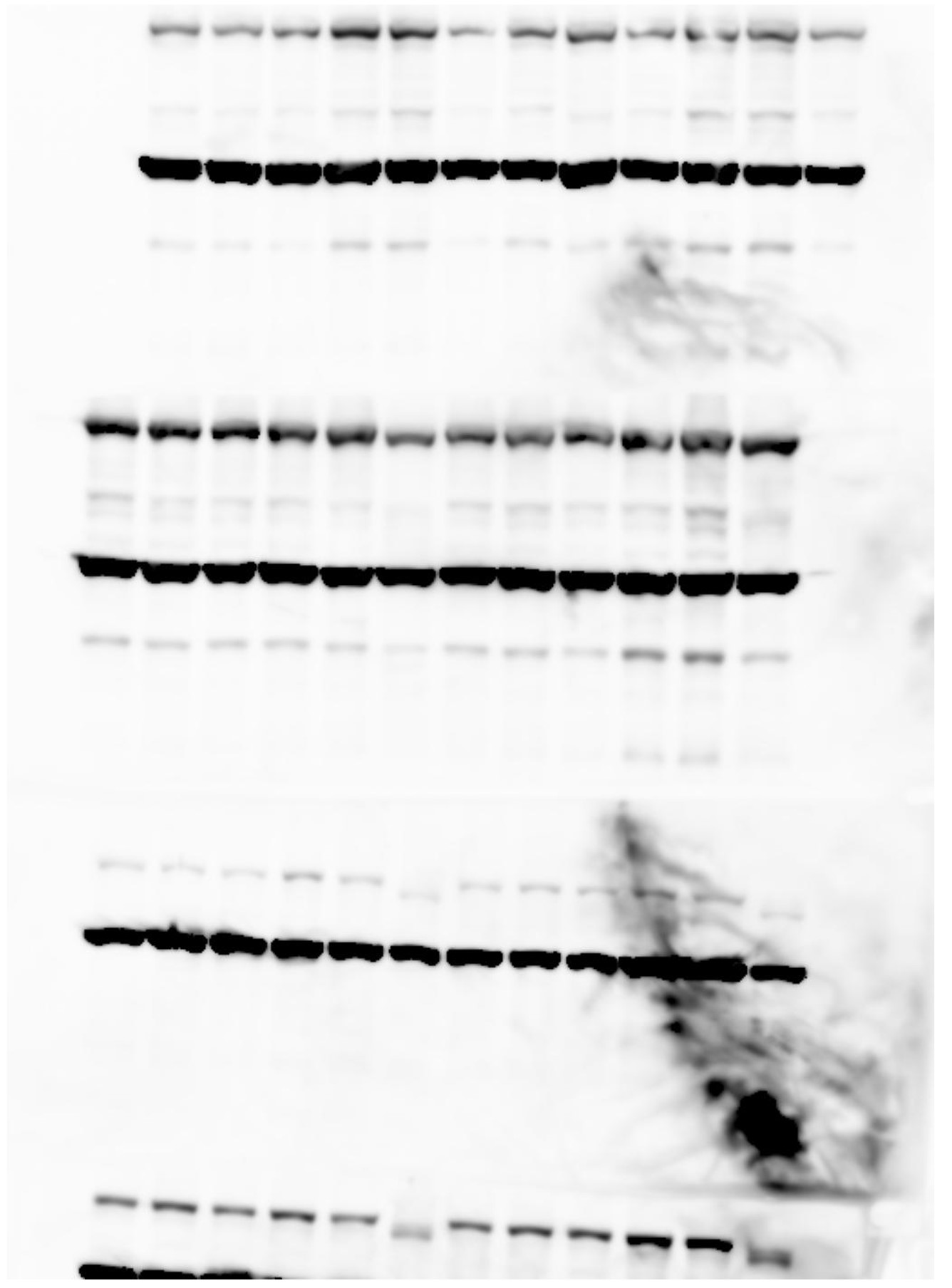

Supplement: Supplementary file 1 [file biomolecules-15-00458-s001.zip › biomolecules-3505096-supplementary new version/File S1/FigS1-6.jpg]
